# New Insights into the Phototoxicity of Anthracene-Based Chromophores: The Chloride Salt Effect†

**DOI:** 10.1021/acs.chemrestox.2c00235

**Published:** 2023-06-22

**Authors:** Mohammad Sadegh Safiarian, Aikohi Ugboya, Imran Khan, Kostiantyn O. Marichev, Kathryn B. Grant

**Affiliations:** Department of Chemistry, Georgia State University, Atlanta, Georgia 30303, United States

## Abstract

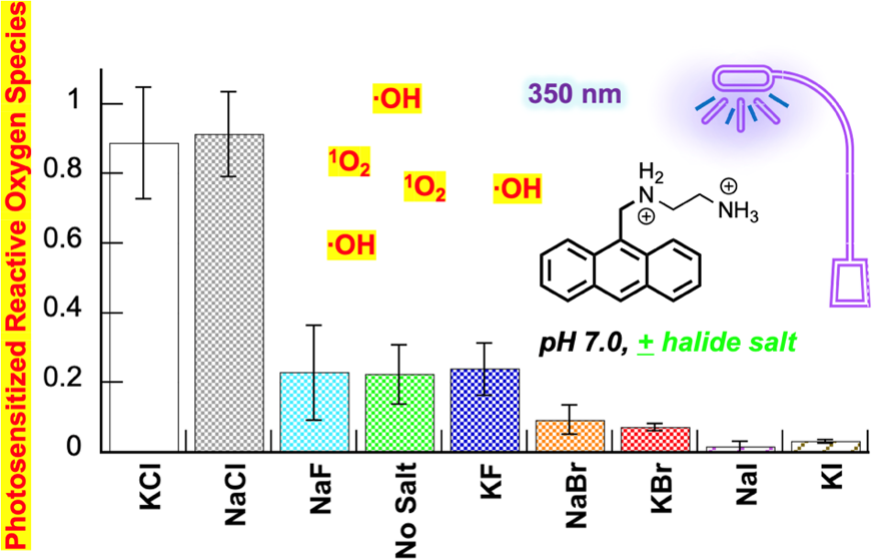

Unraveling the causes underlying polycyclic aromatic hydrocarbon phototoxicity is an essential step in understanding the harmful effects of these compounds in nature. Toward this end, we have studied the DNA interactions and photochemistry of *N*^1^-(anthracen-9-ylmethyl)ethane-1,2-diaminium dichloride in the presence and absence of NaF, KF, NaCl, KCl, NaBr, KBr, NaI, and KI (350 nm hν, pH 7.0). Exposing pUC19 plasmid to UV light in solutions containing 400 mM KCl formed significantly more direct strand breaks in DNA compared to no-salt control reactions. In contrast, NaCl increased DNA damage moderately, while the sodium(I) and potassium(I) fluoride, bromide, and iodide salts generally inhibited cleavage (I^–^ > Br^–^ > F^–^). A halide anion-induced heavy-atom effect was indicated by monitoring anthracene photodegradation and by employing the hydroxyl radical (^•^OH) probe hydroxyphenyl fluorescein (HPF). These studies revealed that among no-salt controls and the eight halide salts, only NaCl and KCl enabled the anthracene to photosensitize the production of high levels of DNA-damaging reactive oxygen species (ROS). Pre-irradiation of *N*^1^-(anthracen-9-ylmethyl)ethane-1,2-diaminium dichloride at 350 nm increased the amounts of chloride salt-induced ^•^OH detected by HPF in subsequent anthracene photoactivation experiments. Taking into consideration that ^•^OH and other highly reactive ROS are extremely short-lived, this result suggests that the pre-irradiation step might lead to the formation of oxidized anthracene photoproducts that are exceedingly redox-active. The fluorometric probes HPF and Singlet Oxygen Sensor Green revealed that KCl concentrations ranging from 150 to 400 mM and from 100 to 400 mM, respectively, enhanced *N*^1^-(anthracen-9-ylmethyl)ethane-1,2-diaminium dichloride photosensitized ^•^OH and singlet oxygen (^1^O_2_) production over no-salt controls. Considering the relatively high levels of Na^+^, K^+^, and Cl^–^ ions that exist in the environment and in living organisms, our findings may be relevant to the phototoxic effects exhibited by anthracenes and other polycyclic hydrocarbons *in vivo*.

## 1 Introduction

Anthracenes, anthraquinones, and other polycyclic aromatic hydrocarbons (PAHs) are notorious environmental pollutants that are mainly generated due to the incomplete combustion of fossil fuels. Despite human actions being a major source, a significant amount of these compounds is released into the environment by volcanoes, oil and coal seepage from their deposits, wildfires, etc.^1,2^ Humans are continually being exposed to anthracenes and other PAHs through different routes. Aside from inhaling PAHs from ambient air and smoking tobacco, PAHs are also present in trace amounts in certain foods as a result of smoking and other forms of processing.^2,3^ A number of PAHs, including anthracene, damage and/or alter DNA and thereby exhibit carcinogenic and/or mutagenic effects in humans.^2,4,5^ The formation of DNA complexes is the primary mechanism of carcinogenesis induced by many PAHs.^2^ In addition to humans, PAHs are highly toxic to living organisms present in soil and aquatic environments. Rain deposits these compounds into the ground, where they find their way into bodies of water and cause harm.

By virtue of their extensively conjugated, aromatic ring systems, many PAHs are robust chromophores that can become more toxic upon exposure to UV irradiation. A main mechanism of phototoxicity is through the generation of reactive oxygen species (ROS) via photodynamic pathways, which are dependent upon the simultaneous exposure of the PAH to ground-state triplet oxygen molecules (^3^O_2_) and UV light. Upon light absorption, the PAHs become elevated in energy to a reactive singlet excited state, and then after intersystem crossing to a longer-lived triplet state, transfer energy or electrons to ^3^O_2_ to form the reactive oxygen species.^6^ For example, a powerful ROS generated within cells is singlet oxygen (^1^O_2_) through Type II energy transfer. A second strongly oxidizing ROS, the hydroxyl radical (^•^OH), is produced via a Type I pathway in which the PAH excited state transfers an electron to ^3^O_2_ either directly or after being converted to an excited state anion radical by an intermediate electron donor. The superoxide anion radical (O_2_^•–^) thus formed then gives rise to hydroxyl radicals through Fenton chemistry.^7^ Both singlet oxygen and hydroxyl radicals are short-lived and highly reactive, capable of oxidizing nearby lipid, nucleic acid, and protein molecules to create cellular and tissue damage that leads to phototoxic injury,^8^ which emerges as acute toxicity, genotoxicity, or both.^9^

The PAH anthracene is a ubiquitous polycyclic aromatic hydrocarbon consisting of three fused benzene rings (Figure S1 in the Supporting Information). It is known to induce phototoxicity in various aquatic organisms from marine bacteria^10^ to zooplankton and algae,^11,12^ different types of fish,^13−15^ fresh water and brine shrimp,^9,16^ clam larvae,^15,17^ and the dragonfly.^18^ It has also been shown that photoinduced toxicity by anthracene can inhibit leaf formation by the duckweed *Lemna gibba*(19,20) and interfere with photosynthesis in plants.^21^ Upon irradiation with UV light, anthracene can cause toxicity not only by photosensitizing ROS production but also by reacting with the ROS to form toxic anthracene photomodification products.^14,21^

Anthraquinones, a major photomodification product of anthracenes, are themselves able to cause significant phototoxicity by generating ROS upon UV irradiation.^21^ A number of anthraquinone derivatives are classified as Group 2A compounds by the International Agency for Research on Cancer (IARC) as probable carcinogens in humans. Interestingly, several plant-derived natural products present in commercially available dietary supplements and cosmetics have anthraquinone structures (Figure S1 in the Supporting Information).^22−24^ For instance, the anthraquinone derivative hypericin is a natural constituent in herbal antidepressant supplements containing St. John’s wort. Hypericin photosensitization generates both singlet oxygen and hydroxyl radicals^22^ and causes skin irritation when patients taking St. John’s wort are exposed to sunlight.^25^ Hypericin has also been reported to effect extensive phototoxic damage to the human lens and retina.^26^*Aloe-emodin*, a trihydroxyanthraquinone, is present in the *Aloe-vera* plant (Figure S1 in the Supporting Information). It is also found in other plants, including leaves of senna (*Cassia angustifolia*) and the rhizome of rhubarb (*Rheum rhaponticum*) that are sold as over-the-counter laxatives.^23^ Upon exposure to UVA, *Aloe-emodin* causes lipid peroxidation and DNA and RNA damage *in vitro*, mostly through the generation of singlet oxygen and hydroxyl radicals.^27^ When Vath and co-workers employed UVA light to irradiate human skin fibroblast cells cultured in the presence and absence of *Aloe-emodin*, cell survival was reduced by ∼100 and 0%, respectively (fluence = 5 J/cm^2^).^28^*Aloe-emodin* can also cause melanoma in mice exposed to UVB light.^29^

**Scheme 1:**
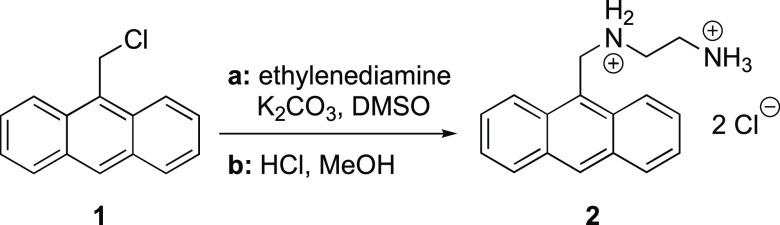
Synthesis of *N*^1^-(Anthracen-9-ylmethyl)ethane-1,2-diaminium Dichloride **2**(30)

In previous papers, we have explored the effects of high-ionic-strength conditions that approximate those that exist in the cell nucleus on oxidative DNA damaged photosensitized by 9-aminomethylanthracene chromophores such as *N*^1^-(anthracen-9-ylmethyl)ethane-1,2-diaminium dichloride (**2** in Scheme 1).^30,31^ The redox-inactive cations Na^+^ and K^+^, respectively, exist in ∼150 and ∼260 mM concentrations in this organelle,^32−34^ where they assist in neutralizing the negatively charged oxygen atoms located in opposing strands of double-helical genomic DNA.^35^ Thanks to their positive charge and their hydrophobic, aromatic natures, the *N*-substituted anthracene derivatives studied in our laboratory were found to interact avidly with nucleic acids and cause extensive photooxidative damage to the DNA under low-ionic-strength conditions. Upon introducing a final concentration of 150 mM NaCl combined with 260 mM KCl into the aqueous solutions containing plasmid DNA and micromolar concentrations of these chromophores, we fully expected that the Na^+^ and K^+^ would reduce 9-aminomethylanthracene-photosensitized DNA damage by effectively competing with the positively charged anthracenes for DNA binding sites. To our surprise, levels of ROS production and concomitant DNA photocleavage were instead enhanced by the high-ionic-strength conditions (350 nm, pH 7.0).^30,31^ Moreover, the phototoxicity of 9-aminomethylanthracene chromophores against *Escherichia coli* was increased by the 150 mM NaCl and 260 mM KCl salt combination.^30^

To gain a better understanding of the mechanisms underlying the unanticipated phenomenon of redox-inactive chloride salts contributing to photooxidative DNA damage, in the current study, we have examined ROS generated by *N*^1^-(anthracen-9-ylmethyl)ethane-1,2-diaminium dichloride **2** when NaCl and KCl are present individually rather than in combination. We have also compared the effects of NaCl and KCl to those of individual sodium(I) and potassium(I) fluoride, bromide, and iodide salts. Taking into consideration the high concentrations of Na^+^, K^+^, and Cl^–^ ions in living cells^32−34,36−39^ and in aquatic environments,^40^ our findings may be relevant to the phototoxic effects exhibited by certain PAHs under physiological conditions.

## 2 Materials and Methods

### 2.1 General

Deionized water was used in the preparation of all solutions. The purity of the halide salts NaF, KF, NaBr, KBr, NaI, and KI was ≥99.99% (Sigma-Aldrich), while NaCl and KCl were trace-metal basis grade (≥99.999% purity; Sigma-Aldrich). Calf thymus DNA (CT DNA; 10 mg/mL, average size ≤ 2000 bp; Cat # 15633019), NBT (nitro blue tetrazolium chloride), Singlet Oxygen Sensor Green (SOSG; Cat # S36002), Tiron (disodium 4,5-dihydroxy-1,3-benzenedisulfonate), and deferoxamine mesylate were from ThermoFisher Scientific. Hydroxyphenyl fluorescein (HPF; 5 mM in DMF) was purchased from Sigma-Aldrich and was used undiluted. Other reagents obtained from Sigma-Aldrich included deuterium oxide, sodium benzoate (99%), ethidium bromide, bovine liver catalase, and 9-(methylaminomethyl)anthracene. The pUC19 plasmid was cloned in *E. coli* DH5α competent cells from ThermoFisher Scientific^41^ and was then isolated and purified using a Qiagen Maxi Kit. DNA concentration and purity were determined by UV–visible spectrophotometry. The known compound *N*^1^-(anthracen-9-ylmethyl)ethane-1,2-diaminium dichloride **2** was synthesized and characterized as reported in our previous publication (Scheme 1).^30^ For long-term storage, 10–15 mM stock solutions of **2** were prepared in dimethyl sulfoxide (DMSO) and kept at −20 °C. From the initial DMSO stock solutions, further anthracene dilutions were made immediately before use, first in pure methanol and from the methanol stock solution, in ddH_2_O. This was done in order to minimize the final concentration of ROS-scavenging DMSO in anthracene reactions.

### 2.2 UV–Visible Spectroscopy

For stability studies, samples containing 50 μM *N*^1^-(anthracen-9-ylmethyl)ethane-1,2-diaminium dichloride **2** and 10 mM sodium phosphate buffer pH 7.0 were prepared in the presence and absence of 400 mM NaF, KF, NaCl, KCl, NaBr, KBr, NaI, and KI. The aqueous samples were then monitored by UV–vis spectroscopy over 1 h to ensure that absorbance did not change over time. For anthracene-DNA interactions experiments, to samples containing 50 μM dye and 400 mM halide salt in 10 mM sodium phosphate buffer pH 7.0, a final concentration of 200 μM bp of CT DNA was added. In order to study the photoinduced degradation of the 9-aminomethylanthracene, solutions containing 50 μM **2** and 10 mM sodium phosphate buffer pH 7.0 in the presence and absence of 400 mM of halide salt were irradiated at time intervals up to 180 s and 5 min inside a ventilated Rayonet photochemical reactor fitted with 8 RPR-3500 Å, 24 W lamps (spectral output ∼310 to 410 nm; The Southern New England Ultraviolet Co.). Absorbance spectra were recorded immediately after irradiation.

### 2.3 Photocleavage of pUC19 DNA

Photocleavage reactions contained 38 μM bp of pUC19 plasmid, 1–10 μM anthracene, and 50–400 mM of a halide salt (NaF, KF, KCl, KCl, NaBr, KBr, NaI, or KI). The samples were prepared in clear microcentrifuge tubes in a total volume of 30 μL, placed in the dark at 25 °C for 1 h to equilibrate, and then irradiated for 1 h inside the ventilated Rayonet photochemical reactor fitted with 8 RPR-3500 Å, 24 W lamps. At the conclusion of reactions, 3 μL of 6X loading buffer (15.0% (*w/v*) Ficoll, 0.025% (*w/v*) bromophenol blue) were added to each of the samples, which were then loaded onto 1.5% nondenaturing agarose gels stained with ethidium bromide (0.5 μg/mL, final concentration). Electrophoresis was done in a Bio-Rad Mini-Sub chamber at 110 V for 1 h. Upon completion, gels were visualized using 302 nm UV light and photographed with a PhotoDoc-It Imaging System (Upland, CA). The pictures taken from gels were quantitated using ImageJ software (National Institutes of Health). Due to the decreased binding affinity of ethidium bromide to the supercoiled versus nicked and linear plasmid forms, the integrated data obtained for supercoiled DNA were multiplied by a correction factor of 1.22.^31^ The percent of DNA cleavage was calculated using the formula: % Cleavage = [(Linear + Nicked DNA)/(Linear + Nicked + Supercoiled DNA)] × 100.

### 2.4 Anthracene and HPF Fluorescence Measurements

Spectroscopic samples consisted of 1 μM anthracene dye **2** or 0.5 μM anthraquinone-2-sulfonate in 10 mM sodium phosphate buffer pH 7.0 with and without 100, 140, 150, and/or 400 mM of a halide salt (either NaF, KF, NaCl, KCl, NaBr, KBr, NaI, or KI). In hydroxyl radical detection experiments, a final concentration of 3 μM of the hydroxyphenyl fluorescein (HPF) probe was added to reactions. The HPF fluorescence samples were transferred to 10 mm × 10 mm quartz cuvettes with four clear sides (2.5 mL total volume each) and were then irradiated for 60, 120, or 420 s at 350 nm inside the Rayonet photochemical reactor. Emission spectra were then recorded. The excitation and emission slit widths were both 4.5 nm. Gain was set at medium. The scan rate was 200 nm/min, and the excitation wavelength was 490 nm for HPF and 366 nm for **2**.

For pre-irradiation experiments, 50 μM stock solutions of **2** in 10 mM sodium phosphate buffer pH 7.0 were transferred to the quartz cuvettes and then pre-irradiated for 0, 60, or 90 s inside the Rayonet photochemical reactor fitted with 8 RPR-3500 Å, 24 W lamps (2.5 mL total volume). Samples containing 1 μM of the pre-irradiated anthracene, 3 μM of the HPF probe, and 10 mM sodium phosphate buffer pH 7.0 were then prepared in the presence and absence of 400 mM KCl (2.5 mL total volume) and irradiated in the cuvettes with the 350 nm UV lamps for 0, 30, 60, or 90 s. HPF emission spectra were then recorded as just described. The area under each HPF curve was measured from 500 to 650 nm and plotted against irradiation time to compare ROS generation by pre-irradiated vs non-pre-irradiated *N*^1^-(anthracen-9-ylmethyl)ethane-1,2-diaminium dichloride **2**.

### 2.5 Singlet Oxygen Detection Using SOSG

A 5 mM solution of SOSG was prepared in methanol on the day of use, stored at −20 °C until the start of an experiment, then discarded after 24 h. Reactions containing 10 mM sodium phosphate buffer pH 7.0, 1 μM anthracene **2**, and 1 μM SOSG in the presence and absence of 100, 140, 150, and 400 mM KCl were prepared (2.5 mL total volume). The samples were kept in the dark or were transferred to 10 mm × 10 mm quartz cuvettes with four clear sides (2.5 mL total volume each) and then irradiated for 30 min at 22 °C in a ventilated Rayonet photochemical reactor equipped with six RPR-3500 Å lamps. Fluorescence emission spectra were immediately recorded from 510 to 650 nm with a PerkinElmer LS55 spectrofluorometer (λ_ex_ = 500 nm). The excitation and emission slit widths were both 4.5 nm. Gain was set at medium. The scan rate was 200 nm/min.

### 2.6 Superoxide Anion Radical Detection Using NBT

The production of anthracene **2** photosensitized superoxide anion radicals was measured with a nitro blue tetrazolium (NBT) assay in which superoxide reduces NBT to the dark blue chromophore formazan.^42^ Solutions containing 10 mM sodium phosphate buffer pH 7.0, 50 μM **2**, and 16 μM NBT in the absence and presence of 100, 140, and 150 mM KCl (450 μL total volume) were either kept in the dark or transferred to 500 mL quartz cuvettes, and then irradiated for 5 min in the ventilated Rayonet Photochemical Reactor equipped with six RPR-3500 Å lamps. A Shimadzu UV–visible spectrophotometer was then used to measure the absorption spectrum of each sample.

### 2.7 Effects of Chemical Additives on ROS Production

Reactions containing 10 mM sodium phosphate buffer pH 7.0 and an ROS-detecting colorimetric or fluorometric probe, either 3 μM HPF, 16 μM NBT, or 1 μM SOSG, were irradiated for 0, 5 (NBT), or 7 min (HPF, SOSG) at 350 nm in the presence and absence of 1 μM **2** (HPF, SOSG) or 50 μM **2** (NBT), 400 mM KCl, and a chemical additive, either 100 mM sodium benzoate, 5 mM deferoxamine mesylate, 1 mM disodium 4,5-dihydroxy-1,3-benzenedisulfonate (Tiron), or 90% *(v*/*v)* D_2_O. Absorption and emission spectra were then recorded as just described.

### 2.8 Effects of Chemical Additives on DNA Photocleavage

Sample reactions containing 2.5 or 10 μM anthracene **2**, 38 μM bp of pUC19 plasmid DNA, 10 mM sodium phosphate buffer pH 7.0, and a chemical additive (a final concentration of either 100 mM sodium benzoate, 100 mM ethylenediaminetetraacetate (EDTA), 10 mM Tiron, 100 U/μL of catalase, or 72% *(v*/*v)* D_2_O) in the presence and absence of 400 mM KCl were prepared. In a set of parallel controls, the chemical additives were replaced by equivalent volumes of ddH_2_O. All samples were preequilibrated in the dark for 1 h at 22 °C and then irradiated for 60 min using a ventilated Rayonet photochemical reactor fitted with six RPR-3500 Å lamps. After the irradiation period, the DNA reactions were electrophoresed on nondenaturing agarose gels, visualized, and quantitated as described in Section 2.3. The percent change in DNA photocleavage caused by each additive was then calculated using the formula: % Change in Cleavage = [(% Total of Linear and Nicked DNA_with additive_ – % Total of Linear and Nicked DNA_without additive_)/(% Total of Linear and Nicked DNA_without additive_)] × 100.

### 2.9 Electrospray Ionization Mass Spectrometry (ESI-MS)

ESI-MS analyses were performed on a Waters Xevo G2-XS Mass Spectrometer (Waters Corporate, Milford, MA) equipped with an electrospray ionization source in positive ion mode. Test samples consisting of 10 mM ammonium formate buffer pH 7.0 and 1 mM *N*^1^*-*(anthracen-9-ylmethyl)ethane-1,2-diaminium dichloride **2** in the presence and absence of 150 mM KCl were irradiated at 350 nm for either 0, 2, 5, or 60 min. The original samples were each combined with a standard solution containing 1 mM acridine and 10 mM ammonium formate buffer pH 7.0 at a ratio of one-to-one and then diluted 100 × with 10 mM of the same buffer. Each sample (5 μL) was then introduced into the ion source through an autosampler with a 200 μL/min flow rate. The instrument operation parameters were optimized as follows: capillary voltage of 1000 V, sample cone voltage of 20 V, desolvation temperature of 350 °C, and a source temperature of 120 °C. Nitrogen was used as cone gas and desolvation gas at pressures of 25 and 800 L/h, respectively. The spectra were acquired through a full scan analysis by Dr. Siming Wang of the Mass Spectrometry Facility at the Georgia State University Department of Chemistry. MassLynx 4.2 software was used for data acquisition and processing.

## 3 Results and Discussion

### 3.1 Anthracene-Photosensitized ROS

In previously published work,^30^ we described the synthesis of *N*^1^*-*(anthracen-9-ylmethyl)ethane-1,2-diaminium dichloride (**2**) (Scheme 1). Using the fluorometric, hydroxyl radical probe hydroxyphenyl fluorescein (HPF) and ROS scavengers such as sodium benzoate, we then demonstrated that this chromophore photosensitized the production of high levels of DNA-damaging hydroxyl radicals in the presence of 150 mM NaCl in combination with 260 mM KCl, salts which we used to approximate Na^+^ and K^+^ concentrations typical of those found in the cell nucleus.^32−34^

**Figure 1:**
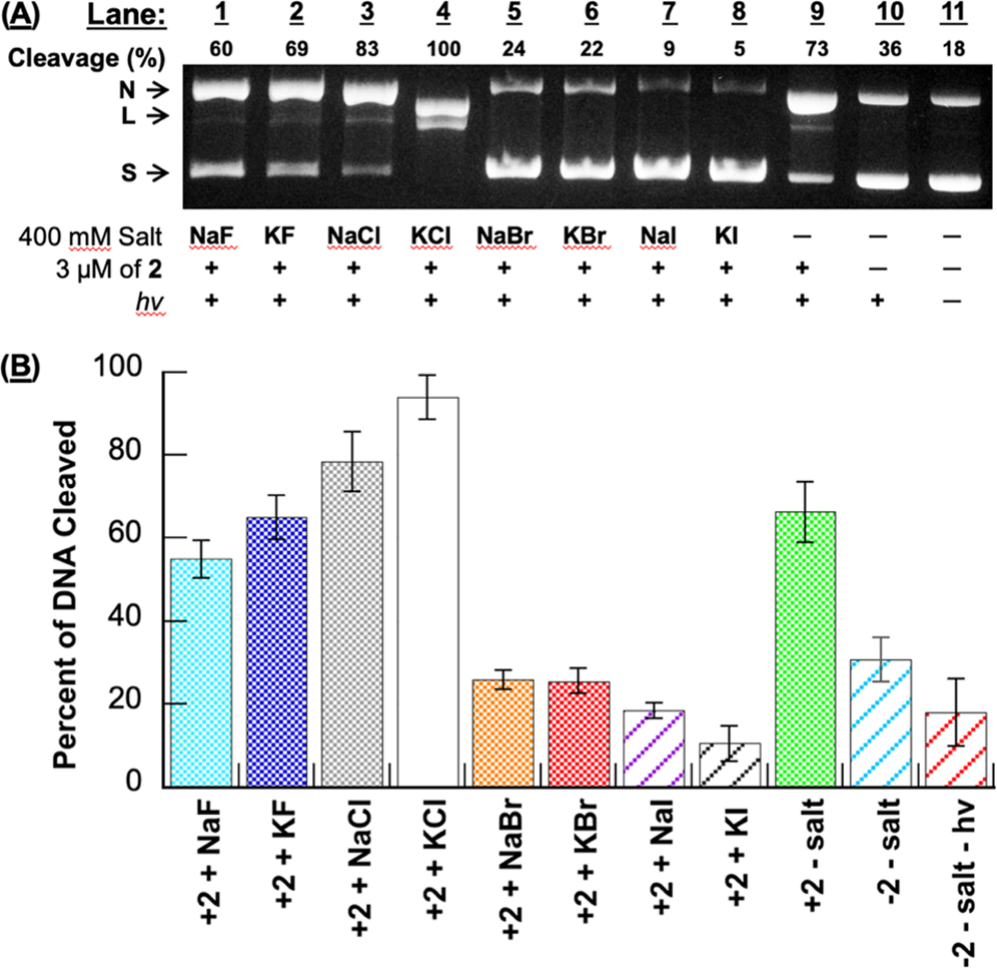
(A) Photograph of a representative 1.5% nondenaturing agarose gel showing photocleavage of 38 μM bp pUC19 plasmid DNA in the presence of 3 μM *N*^1^*-*(anthracen-9-ylmethyl)ethane-1,2-diaminium dichloride 2 without and with 410 mM of halide salt (10 mM sodium phosphate buffer pH 7.0). The samples in Lanes 1 to 10 were irradiated for 60 min in a ventilated Rayonet photochemical reactor fitted with 8 RPR-3500 Å, 24 W lamps. Abbreviations: L = linear; N = nicked; S = supercoiled. (B) Percentage of cleaved pUC19 (linear DNA + nicked DNA) in the photocleavage reactions averaged over 3 trials. Error bars represent standard deviation.

**Figure 2:**
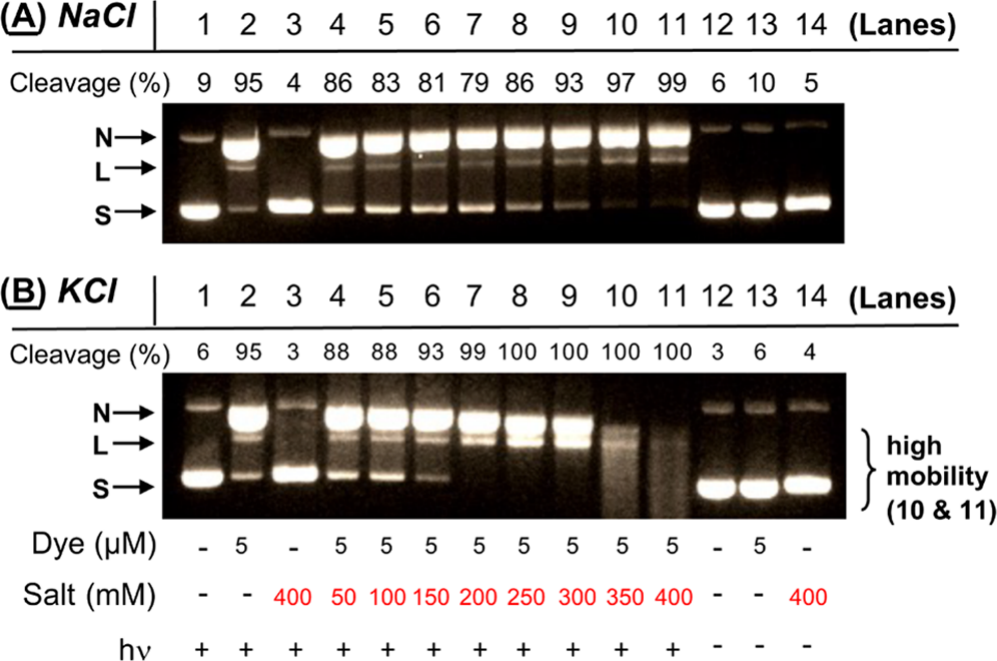
Photographs of 1.5% nondenaturing agarose gels showing photocleavage of pUC19 plasmid DNA in the presence of 5 μM *N*^1^*-*(anthracen-9-ylmethyl)ethane-1,2-diaminium dichloride **2** and concentrations of (A) NaCl and (B) KCl ranging from 0 to 400 mM. Samples contained 10 mM sodium phosphate buffer pH 7.0 and 38 μM bp DNA. The reactions in Lanes 1 to 11 were irradiated at 350 nm for 60 min in a ventilated Rayonet photochemical reactor fitted with 8 RPR-3500 Å, 24 W lamps. In Lanes 10 and 11 of (B), the plasmid was photodegraded into a diffuse band of low-molecular-weight DNA fragments.^30 ,31^ Percent cleavage yields reflect linear, nicked, and fragmented DNA. Abbreviations: L = linear; N = nicked; S = supercoiled.

In our first experiments in the present study, we have accessed contributions from individual sodium(I) and potassium(I) fluoride, chloride, bromide, and iodide salts on the formation of anthracene **2-** photosensitized DNA direct strand breaks. Toward this end, reactions containing 3 μM **2**, 38 μM bp of pUC19 plasmid DNA, 10 mM sodium phosphate buffer pH 7.0, and 410 mM of a halide salt, either NaF, KF, NaCl, KCl, NaBr, KBr, NaI, or KI were irradiated at 350 nm for 60 min in a ventilated Rayonet photochemical reactor. DNA reaction products were then resolved and visualized on nondenaturing agarose gels. The data in Figure 1 show the resulting anthracene-sensitized photoconversion of uncut supercoiled plasmid to nicked and linear DNA forms. In this figure, a typical gel is placed above a histogram that illustrates the averaged percent of nicked + linear DNA in photocleavage reactions run over three gel trials. A robust DNA direct strand break enhancement effect is observed for KCl and for NaCl, albeit to a lesser extent (Lanes 4 and 3 in Figure 1A compared to the no-salt control in Lane 9). In contrast, the sodium(I) and potassium(I) bromide and iodide salts are shown to strongly reduce cleavage yields, while weak inhibition is exhibited in the case of NaF. Overall, the data in Figure 1 demonstrate that *N*^1^*-*(anthracen-9-ylmethyl)ethane-1,2-diaminium dichloride **2** photosensitizes DNA direct strand breaks in the order: KCl ≫ NaCl ≥ no-salt ≈ KF > NaF ≫> NaBr ≈ KBr > NaI > KI. The agarose gel in Figure 1A reveals that KCl is the only salt capable of promoting significant cleavage of nicked plasmid into the linear DNA form. It is important to take note that the DNA photocleavage yields reported in Figure 1B and elsewhere in this paper do not distinguish between nicked, linear, and low-molecular-weight fragmented plasmid forms. In this regard, the yields can underestimate the effects of KCl on the magnitude of DNA cleavage (*e.g.*, Figure 2B, Lanes 8 to 11).

**Figure 3:**
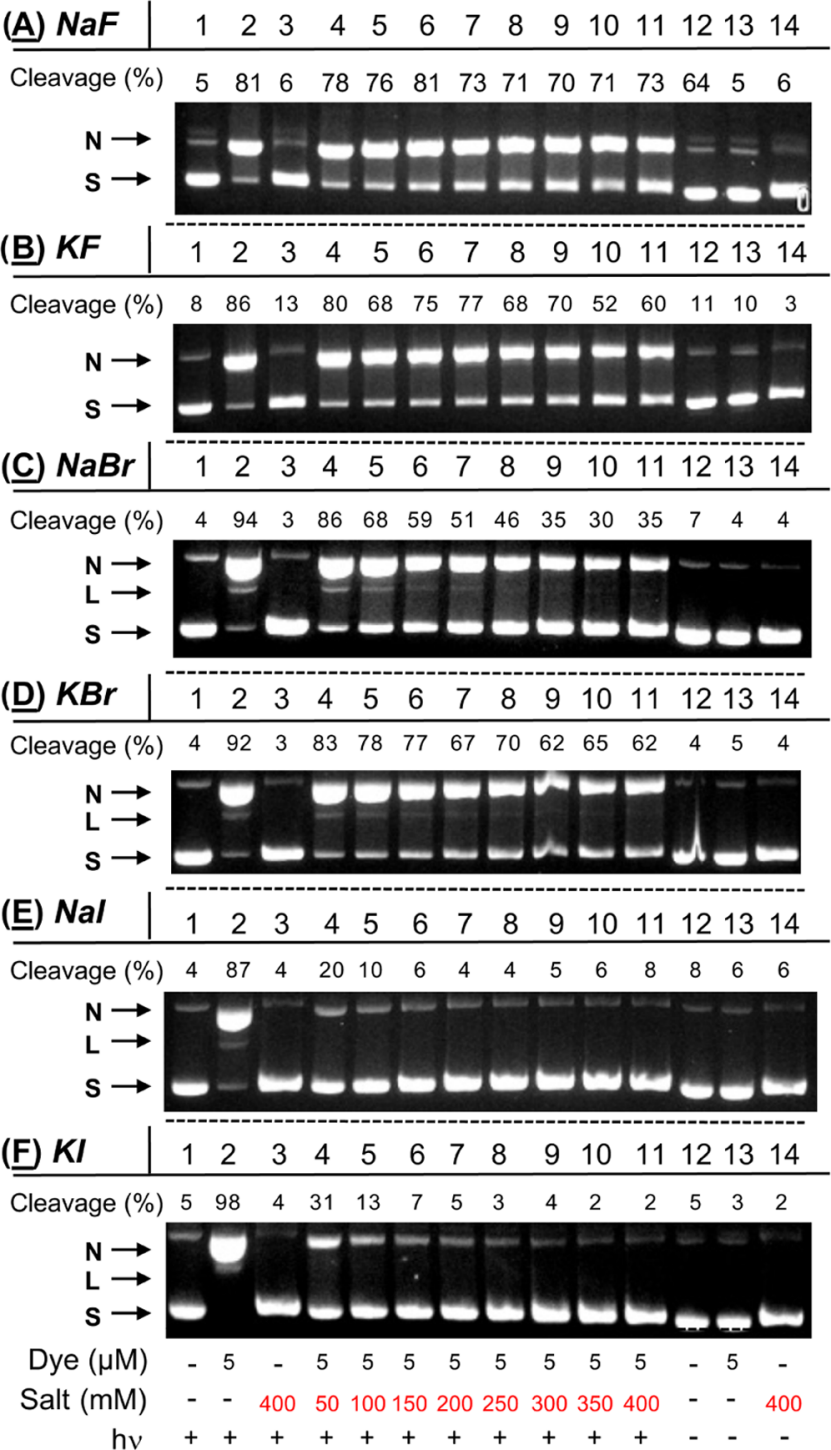
Photographs of 1.5% nondenaturing agarose gels showing photocleavage of pUC19 plasmid DNA in the presence and absence of 5 μM *N*^1^*-*(anthracen-9-ylmethyl)ethane-1,2-diaminium dichloride **2** and 0 to 400 mM of (A) NaF, (B) KF, (C) NaBr, (D) KBr, (E) NaI, and (F) KI. Samples contained 10 mM sodium phosphate buffer pH 7.0 and 38 μM bp DNA. The reactions in Lanes 1 to 11 were irradiated at 350 nm for 60 min. Percent cleavage yields reflect linear and nicked DNA. Abbreviations: L = linear; N = nicked; S = supercoiled.

In our next experiment, we monitored the formation of *N*^1^*-*(anthracen-9-ylmethyl)ethane-1,2-diaminium dichloride **2** photosensitized direct strand breaks as a function of increasing ionic strength, from 50 mM up to 400 mM for each of the eight halide salts (Figures 2 and 3). At low KCl concentrations, DNA photocleavage levels were slightly inhibited (Lanes 4 and 5 in Figure 2B) compared to the no-salt control reaction in Lane 2. However, KCl then generated very pronounced cleavage enhancements starting at 150 mM of salt up to 400 mM (Lanes 6 through 11), with DNA being heavily degraded into diffuse bands of low-molecular-weight fragments at 350 and 400 mM KCl (Lanes 10 and 11 in Figure 2B). In contrast, NaCl concentrations above 300 mM caused moderate increases in cleavage, while 50 to 200 mM of salt resulted in inhibition (Figure 2A). In the case of NaF, KF, NaBr, KBr, NaI, and KI, the overall net effect produced by adding more salt was to incrementally reduce DNA cleavage yields relative to the Lane 2 no-salt controls, although one or more outlying data points were observed in each of the salt series (Figure 3). Extremely low levels of cleaved DNA were seen when solutions containing 400 mM of the salt and anthracene **2** were kept in the dark (Figure S2 in the Supporting Information).

**Figure 4:**
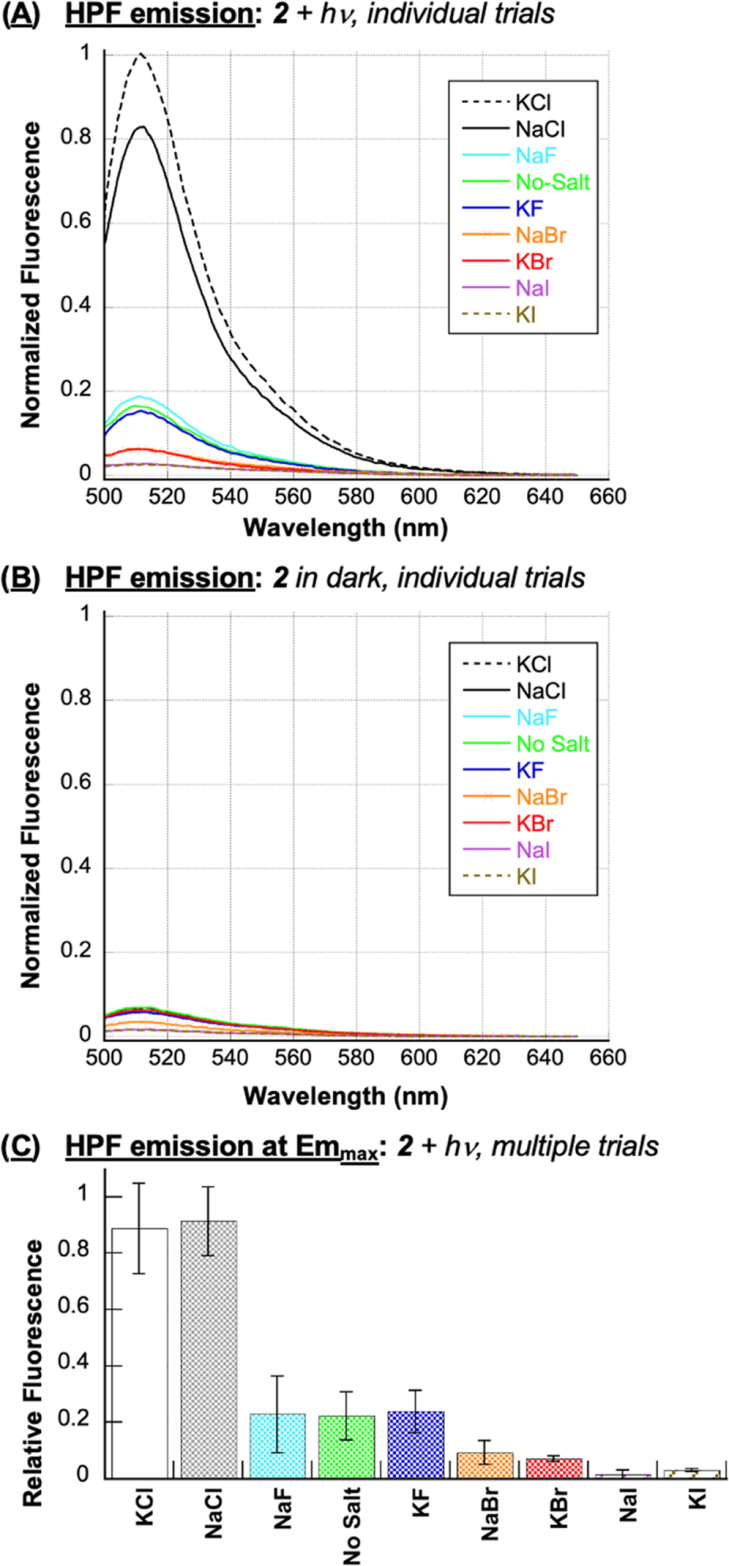
Representative, normalized HPF emission spectra of runs in the (A) presence and (B) absence of 400 mM NaF, KF, NaCl, KCl, NaBr, KBr, NaI, or KI (no DNA). The samples, which consisted of 1 μM *N*^1^*-*(anthracen-9-ylmethyl)ethane-1,2-diaminium dichloride **2**, 3 μM HPF, and 10 mM sodium phosphate buffer pH 7.0, were irradiated for 120 s in a ventilated Rayonet photochemical reactor fitted with 8 RPR-3500 Å, 24 W lamps. HPF emission spectra were then recorded from 500 to 650 nm at an excitation wavelength of 490 nm. (C) Normalized fluorescence emission at 511.5 nm from individual runs averaged over two to three trials. Error bars represent standard deviation.

Upon reaction with ROS, the hydroxyphenyl unit of hydroxyphenyl fluorescein (HPF) is released, and the remaining fluorescein moiety shows increased fluorescence at 512 nm.^43^ HPF primarily detects hydroxyl radicals (^•^OH) and is significantly less reactive toward other ROS, sensing ^•^OH, superoxide anions radicals (O_2_^•–^), singlet oxygen (^1^O_2_), and hydrogen peroxide (H_2_O_2_) at ratios of 730:8:5:2 (sodium phosphate buffer pH 7.4).^44^ When we previously irradiated anthracene **2** in D_2_O, a solvent that increases the lifetime of singlet oxygen approximately 10-fold,^45^ fluorescence emission was slightly decreased relative to parallel H_2_O controls,^30^ suggesting that HPF would be a good probe to confirm ^•^OH production in our current experiments (Figures 1–3). Toward this end, anthracene **2** and HPF were irradiated at 350 nm for 120 s in the presence of 400 mM of each halide salt (22 °C, pH 7.0; Figure 4). Since nucleic acids react avidly with hydroxyl radicals, we considered the DNA to be a scavenging agent and omitted it from the reactions. As expected, irradiated control samples containing the HPF and the individual halide salts in the absence of anthracene did not generate a significant increase in the HPF signal at 512 nm (Figure S3 in the Supporting Information). In contrast, the fluorometric data obtained in the presence of *N*^1^*-*(anthracen-9-ylmethyl)ethane-1,2-diaminium dichloride **2** showed very high HPF fluorescence signals for KCl as well as NaCl, and much weaker emission in the case of the remaining 6 halide salts: KCl ≈ NaCl ≫≫ no-salt ≈ KF ≈ NaF > NaBr ≈ KBr > NaI ≈ KI (Figure 4B). With KCl as a notable exception, the relative levels of ROS detected by HPF were in general agreement with DNA photocleavage yields (Figure 1: KCl ≫ NaCl ≥ no-salt ≈ KF > NaF ≫> NaBr ≈ KBr > NaI > KI).

To aid in the interpretation of the HPF data in Figure 4, absorption spectra of *N*^1^*-*(anthracen-9-ylmethyl)ethane-1,2-diaminium dichloride **2** were recorded in the presence of 400 mM of each of the eight halide salts (10 mM sodium phosphate buffer pH 7.0, no DNA). Figure S4 shows that anthracene **2** absorbs similar amounts of light and is stable over the course of an hour, irrespective of which salt is present. Thus, major changes in anthracene absorption were likely not to be a major factor influencing the differential ROS levels observed in the HPF experiments shown in Figure 4.

**Figure 5:**
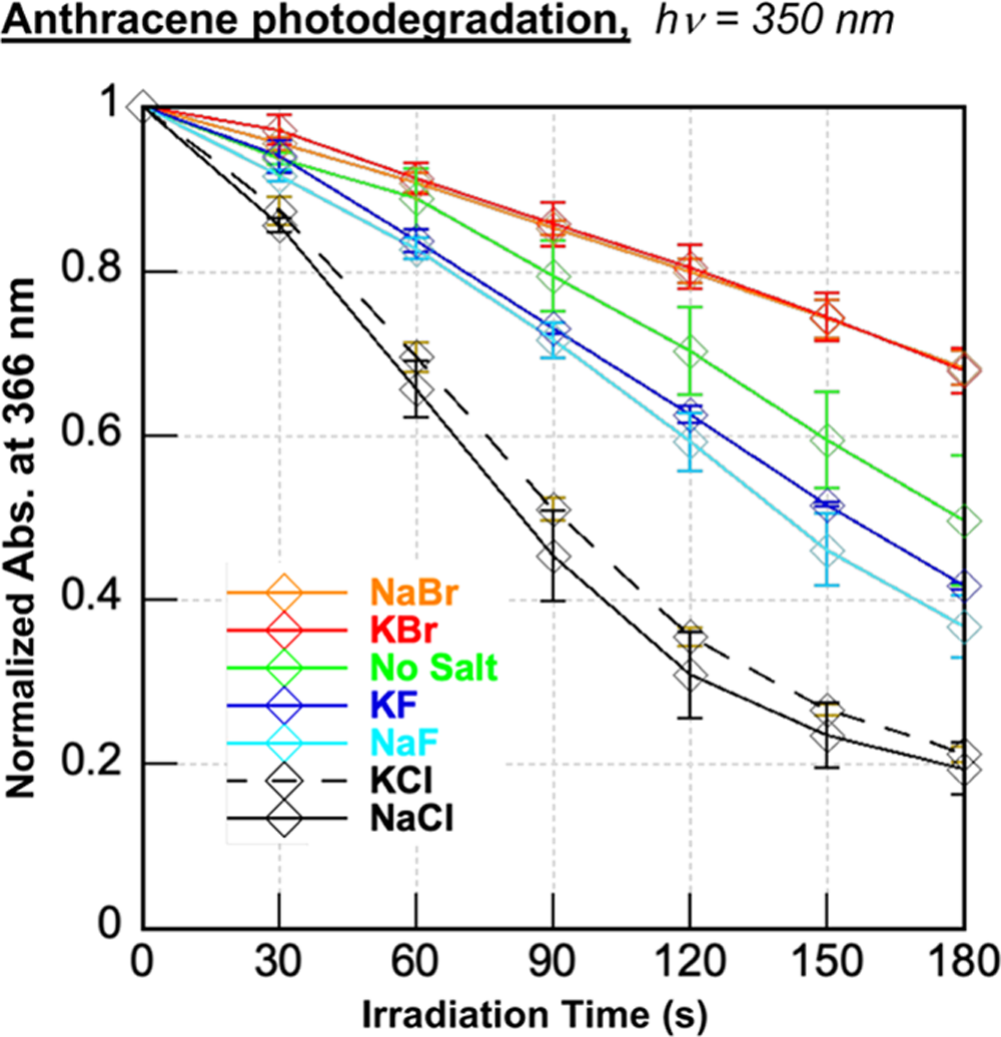
Photobleaching experiments in which averaged, normalized anthracene absorption at 366 nm is plotted as a function of irradiation time. Samples containing 50 μM **2** in the presence and absence of 400 mM of individual halide salts (10 mM sodium phosphate buffer pH 7.0) were irradiated in a ventilated Rayonet photochemical reactor fitted with 8 RPR-3500 Å, 24 W lamps. The averaged data were obtained from normalized absorption spectra recorded over three trials. Error bars represent standard deviation.

Upon absorption of a photon of light, polycyclic aromatic hydrocarbons become oxidized by the ROS that they generate upon reaction of the PAHs’ triplet states with ground-state triplet oxygen, both singlet oxygen from energy transfer and hydroxyl radicals produced via electron transfer.^14,21,46,47^ This process, called photodegradation, generally alters PAH absorption. Interestingly, increasing the concentration of NaCl in estuarine waters has been shown to enhance the photodegradation rates of various PAHs (*e.g.*, carbamazepine, phenanthrene, benzo(a)pyrene, and benzo(e)pyrene) by enriching the production of PAH-photosensitized radical species.^47,48^ To confirm the ROS trends suggested by our preceding DNA photocleavage and HPF experiments (Figures 2–4), we accordingly monitored anthracene photodegradation (a.k.a., photomodification) by recording absorption spectra of **2** after irradiation with a 350 nm UV light source for 30 s time intervals up to 180 s (no DNA; Figure S5). The absorbance of **2** at 350 nm was then plotted as a function of irradiation time (Figure 5). In good general agreement with the HPF assay in Figure 4, the photobleaching experiments suggested that the highest levels of ROS were formed when either 400 mM sodium or potassium chloride was present. Photoinduced degradation of the anthracene was observed with sodium and potassium chloride followed by fluoride salts, no-salt conditions, and bromide salts: KCl ≈ NaCl ≫> NaF ≈ KF > no-salt > KBr ≈ NaBr. Iodide salt data could not be interpreted because, upon irradiation, sample turbidity distorted absorption measurements (Figure S5). Also, in agreement with the HPF data in Figure 4, the photobleaching experiments demonstrated that the ability of NaCl and KCl to enhance anthracene-photosensitized ROS production is independent of DNA. Thus, it is not necessary for DNA to serve as an anthracene triplet state electron donor for effective salt-dependent ROS photosensitization.

**Figure 6:**
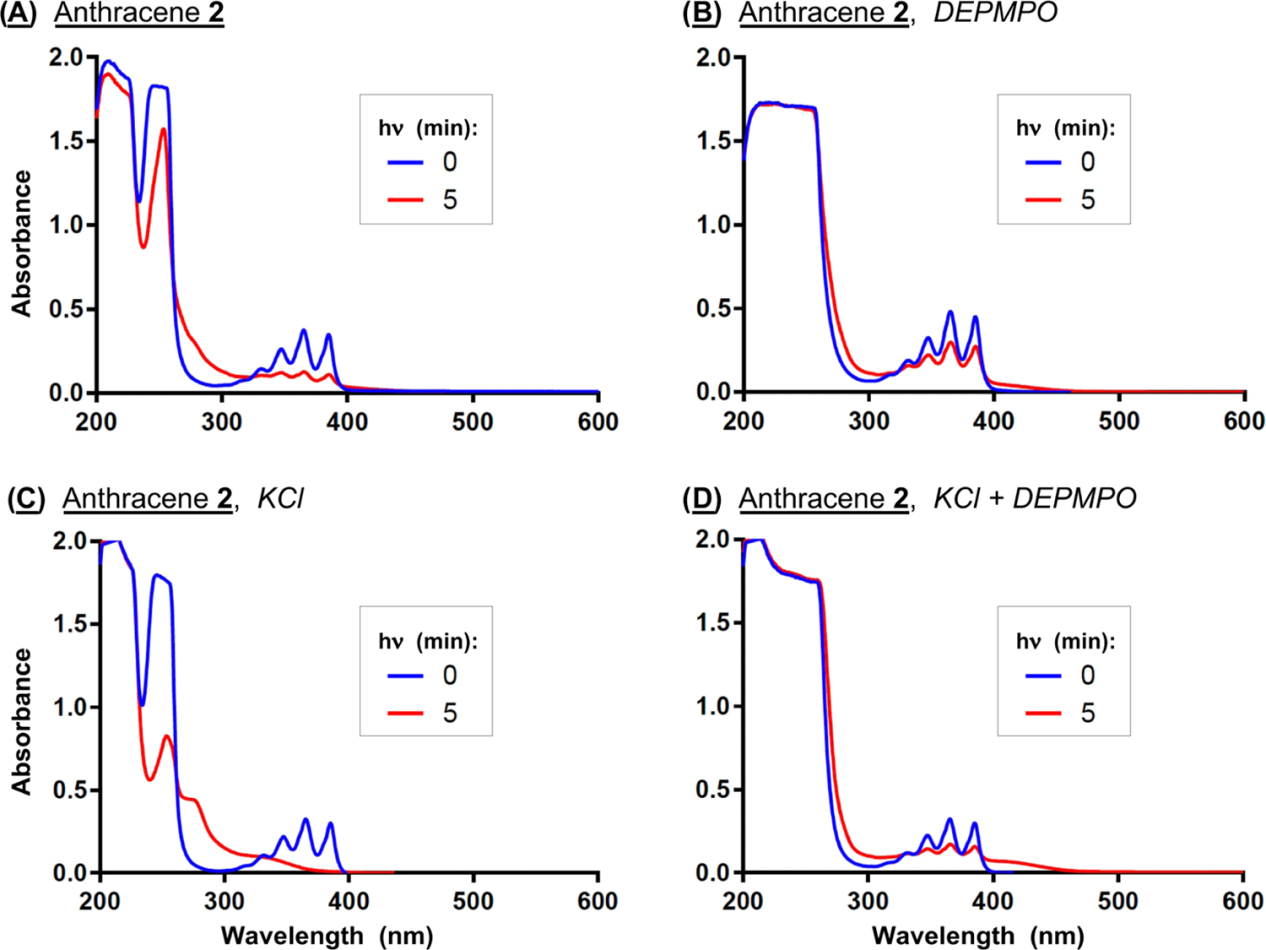
(A–D) Absorption spectra of 50 μM **2** in the presence and absence of 400 mM KCl and 5 mM DEPMPO after irradiation for 0 and 5 min (10 mM sodium phosphate buffer pH 7.0; 350 nm).

In order to confirm the involvement of reactive oxygen species in anthracene **2** photodegradation, we employed 5-diethoxyphosphoryl-5-methyl-1-pyrroline-*N*-oxide (DEPMPO), a spin trap that forms adducts with a number of oxygen-centered radicals, including hydroxyl radicals and their superoxide anion radical precursors.^49^ Solutions composed of the anthracene in the presence and absence of 400 mM KCl and DEPMPO were irradiated at 350 nm for 0 and 5 min and then analyzed by UV–visible spectrophotometry. The spectra in Figure 6 show that KCl markedly enhances anthracene photodegradation, which is associated with a reduction in absorption of the vibrational bands of **2** accompanied by increased hypsochromic absorption between ∼ 280 and ∼320 nm. The addition of the DEPMPO spin trap to the no-salt and KCl reactions attenuates these UV-induced absorption changes, suggesting that ROS photosensitized by anthracene **2** contribute to photodegradation.

In our DNA photocleavage experiments, KCl and NaCl were the only two among eight halide salts to enhance levels of *N*^1^*-*(anthracen-9-ylmethyl)ethane-1,2-diaminium dichloride **2-**sensitized DNA direct strand breaks over no-salt controls (Figures 1–3). Cleavage yields were highest in the presence of 400 mM of the chloride salts, followed by their fluoride, bromide, and iodide counterparts (Figures 1 and 2). In the HPF and photodegradation assays, which were conducted in the absence of DNA, the halide salts KCl and NaCl were also associated with much higher levels of anthracene-photosensitized ROS production (Figures 4 and 5). Rather than KCl being superior to NaCl, as was the case in DNA photocleavage (Figures 1 and 2), the amounts of reactive oxygen species generated in the presence of the two chloride salts were equivalent within experimental error (Figures 4 and 5). Notwithstanding, when taken together, the DNA photocleavage, HPF, and photodegradation data show that chloride anions play a major role in influencing *N*^1^*-*(anthracen-9-ylmethyl)ethane-1,2-diaminium dichloride **2**-sensitized ROS production.

### 3.2 Heavy-Atom Effect

The primary mechanism of photoinduced toxicity and ROS generation by PAHs is through the excited triplet state transfer of electrons (Type I) or energy (Type II) to ground-state triplet oxygen.^6^ EPR analyses of triplet anthracene in frozen aqueous micellar solutions have estimated the triplet state lifetimes and yields of anthracene in the presence of halogenated micelles. As triplet lifetimes decreased in the order 37.5 ms (F) > 34.2 ms (Cl) > 20.4 ms (Br) > 8.1 ms (I), triplet state yields increased as follows: 10.2 (F) < 33.2 (Cl) < 40.2 (Br) < 42 (I). These studies strongly point to a heavy-atom effect that accelerates intersystem crossing from the singlet excited state S_1_ of anthracene to the triplet excited state T_1_, with the heavier Br^–^ and I^–^ anions causing subsequent triplet state quenching via a T_1_ to S_0_ transition.^50^ In work by Kahan et al., halide salts were shown to increase the photodegradation rates of anthracene in aqueous solutions in the order NaCl > NaBr > NaI. The rate enhancements were attributed by the authors primarily to a heavy-atom effect involving singlet oxygen production from anthracene’s triplet excited state.^51^

**Figure 7:**
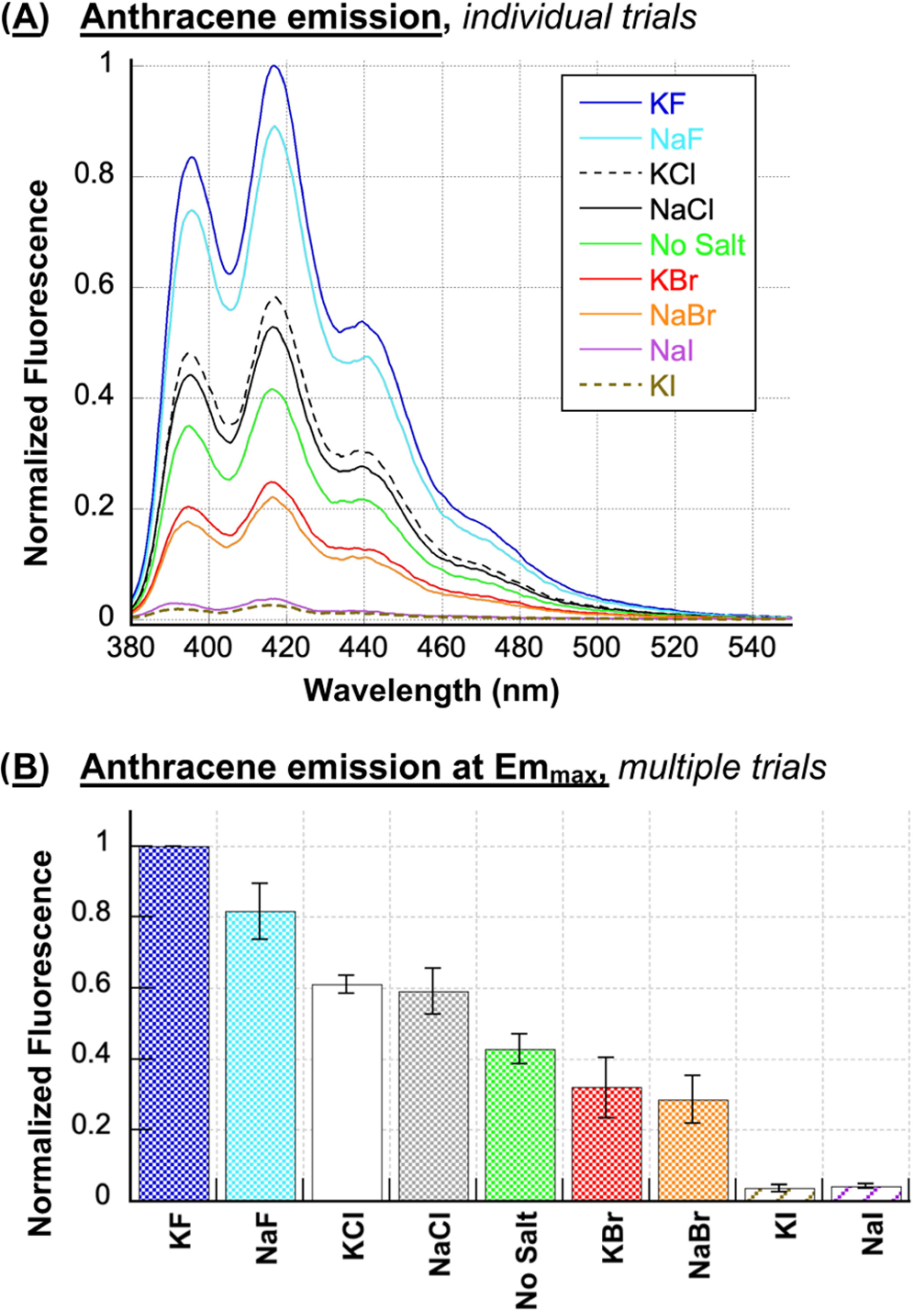
(A) Representative, normalized fluorescence emission spectra of 1 μM *N*^1^*-*(anthracen-9-ylmethyl)ethane-1,2-diaminium dichloride **2** in the presence and absence of 400 mM NaF, KF, NaCl, KCl, NaBr, KBr, NaI, or KI (10 mM sodium phosphate buffer pH 7.0; no DNA). The spectra were recorded from 380 to 550 nm using an excitation wavelength of 366 nm. (B) Normalized fluorescence at the Em_max_ of the anthracene (416.5 ± 0.5 nm) averaged over three trials. Error bars represent standard deviation.

In the current investigation, indirect evidence suggestive of a heavy-atom effect was obtained by acquiring fluorescence spectra of *N*^1^*-*(anthracen-9-ylmethyl)ethane-1,2-diaminium dichloride **2** with and without the halide salts (no DNA, Figure 7). The observed ordering of fluorescence intensities (F^–^ > Cl^–^ > Br^–^ > I^–^) could be the result of heavy-atom-mediated intersystem crossing from the singlet excited state S_1_ to the triplet excited state T_1_ of the anthracene. It is possible that the high levels of anthracene-photosensitized ROS production that occur when either KCl or NaCl is present in our experiments might result from chloride striking an optimal balance between triplet state lifetimes and yields compared to fluoride, bromide, and iodide anions.

### 3.3 Hydroxyl Radicals vs Singlet Oxygen

**Scheme 2:**
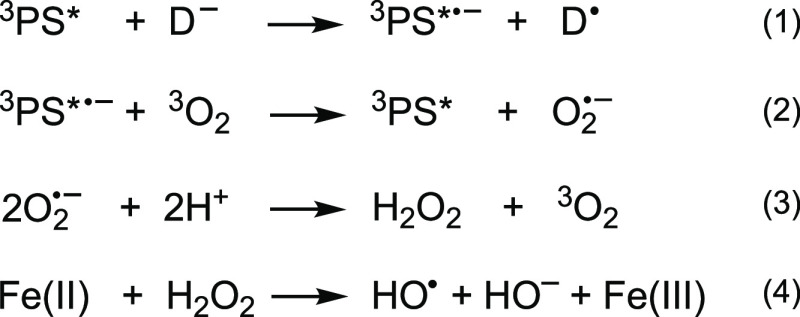
Proposed Mechanism for Hydroxyl Radical Generation via Electron Transfer from the Triplet Excited State of a Photosensitizer (PS) to Ground-State Triplet Oxygen ^3^O_2_

In addition to Type II singlet oxygen (^1^O_2_), anthracene exposed to sunlight generates hydroxyl radicals (^•^OH) that cause DNA damage and cell cycle arrest.^52^ The formation of ^•^OH involves an initial donation of an electron from the solvent or other donor molecule (D^–^) to the triplet excited state of anthracene to form an excited state anion radical (^3^PS*^•–^; Scheme 2). This is followed by Type I transfer of an electron from ^3^PS*^•–^ to ground-state triplet oxygen (^3^O_2_).^52^ The superoxide anion radicals (O_2_^•–^) thus produced in turn spontaneously dismutate at neutral pH^53,54^ to generate hydrogen peroxide, which gives rise to hydroxyl radicals through an Fe^2+^ (or Cu^+^)-based Fenton reaction.^7^ The ^1^O_2_ and ^•^OH produced upon chromophore photoactivation are both highly reactive oxidizing agents, with estimated, respective diffusion distances and half-lives of 50–100 nm^55^ and 3 μs^56^ (for ^1^O_2_); 0.8–6.0 nm^57^ and 1 ns^58^ (for ^•^OH). By comparison, the superoxide anion radical, which accelerates Fenton Chemistry by reducing Fe(III) to Fe(II), has a half-life of approximately 5 s at physiological pH.^59^

There is unanimity among scholars that hydroxyl radicals generate DNA direct strand breaks in a non-sequence-specific fashion by abstracting hydrogen atoms from 2-deoxyribose,^7^ while singlet oxygen forms 8-hydroxy-2′-deoxyguanosine lesions and other oxidation products that are minimally labile.^60^ A number of research groups including ours have addressed the issue of ^1^O_2_ reactivity by utilizing D_2_O, a solvent that increases the lifetime of singlet oxygen approximately 10-fold.^45^ When D_2_O has been used to replace H_2_O in photocleavage experiments involving a wide range of chromophores, substantial increases in direct strand break formation have been reported for plasmid DNA reactions resolved on nondenaturing agarose gels.^31,61−65^ These results lend strong support to our hypothesis that ^1^O_2_ is indirectly involved in the formation of DNA direct strand breaks.

This being said, in order to test the Type I electron transfer mechanism put forward in Scheme 2 as well as to probe for Type II singlet oxygen, reactions containing 10 μM anthracene **2** and pUC19 plasmid DNA in the absence and presence of reaction-targeting chemical additives including D_2_O were irradiated at 350 nm and then run on nondenaturing agarose gels. The resulting percent change in total photoinduced direct strand breaks caused by each added reagent was calculated over multiple trials (10 μM **2**; footnote ^b^ in Table 1) and compared to previously published experiments in which we employed a lower concentration of **2** (1 μM; footnote ^d^ in Table 1).^30^ The chelating agent EDTA, Tiron,^66^ the reducing enzyme catalase, and sodium benzoate were respectively employed to test for the participation of metal ions, superoxide anion radicals, hydrogen peroxide, and hydroxyl radicals. To determine if anthracene **2** sensitizes the production of DNA-damaging ^1^O_2_, parallel photocleavage reactions were run in H_2_O and D_2_O. As just mentioned, a DNA cleavage enhancement in the presence of D_2_O would point to Type II singlet oxygen involvement, while the inhibition of DNA direct strand breaks by EDTA, Tiron, catalase, and sodium benzoate would support Type I electron transfer (Scheme 2).

**Table 1:** Average % Change in DNA Photocleavage Induced by Scavengers and D_2_O^a^^e^

reagents (target)	EDTA (M^n+^)	Na benzoate (^•^OH)	Tiron (O_2_^•–^)	D_2_O (^1^O_2_)	catalase (H_2_O_2_)
**2** no-salt^b^	–52 ± 8	–30 ± 13	–61 ± 4	–1 ± 1	+6 ± 6
**2** salt^b^	–46 ± 7	–40 ± 4	–74 ± 4	+4 ± 4	+2 ± 2
**2** no-salt^c^	nd	nd	nd	nd	+10 ± 1
**2** salt^c^	nd	nd	nd	nd	–26 ± 4
**2** no-salt^d^	nd	–48 ± 6	nd	–21 ± 3	+13 ± 8
**2** salt^d^	nd	–85 ± 3	nd	+1 ± 2	–68 ± 3

a Photocleavage reactions consisted of 38 μM bp of pUC19 plasmid DNA equilibrated with 10 mM sodium phosphate buffer pH 7.0.

b 10 μM**2**, and either 100 mM EDTA or Na benzoate, 10 mM Tiron, 100 U/μL of catalase, or D_2_O (72% final volume) in the absence and presence of 400 mM KCl.

c 2.5 μM**2** and 100 U/μL of catalase in the absence and presence of 400 mM KCl.

d 1 μM**2**, and either 100 mM scavenger, 100 U/μL of catalase, or D_2_O in the absence and presence of 150 mM NaCl and 260 mM KCl.

e ^b–d^Samples were aerobically irradiated for 60 min at 350 nm. Data were averaged over three to five trials with error reported as standard deviation.^d^Data in row previously published.^30^

The results of the EDTA, Tiron, and sodium benzoate experiments in Table 1 suggest that redox-active metal ions (*e.g.*, Fe^2+^, Cu^+^), superoxide anion radicals, and hydroxyl radicals contribute to the formation of anthracene **2** photosensitized direct DNA strand breaks irrespective of the ionic strength. In contrast, the reactivity of catalase changed dramatically based on reaction conditions. In the presence of 10 μM **2**, DNA photocleavage was enhanced with and without KCl (footnote ^b^ in Table 1). However, when 400 mM KCl was present, catalase progressively inhibited strand scission as the concentration of **2** was lowered to 2.5 μM (footnote ^c^ in Table 1) and then to 1 μM (footnote ^d^ in Table 1). This suggested to us that hydrogen peroxide might be an intermediate leading to hydroxyl radical formation when KCl is included in anthracene reactions. We reasoned that the hydroxyl radical levels generated at 10 μM anthracene might be high enough to damage the enzyme catalase, preventing it from reducing hydrogen peroxide to water. Thus, lowering amounts of anthracene **2** would be likely to increase catalase activity (Table 1). Overall, the chemical additive experiments on nondenaturing agarose gels support the Type I mechanism proposed in Scheme 2 for anthracene **2** reactions containing KCl. The source of the adventitious levels of Fe^2+^ and/or Cu^+^ that gave rise to the formation of Type I anthracene **2** photosensitized hydroxyl radicals in these experiments is unknown, however. While the employed NaCl and KCl salts were trace-metal basis grade (purity ≥ 99.999%), minute amounts of metal ions could have been introduced into our reactions through ddH_2_O,^67^ laboratory plasticware,^68^ and even sequestered by metal binding sites in our pUC19 DNA upon the alkaline lysis step of plasmid purification from the *E. coli* host cells. This being said, trace levels of copper and iron ions are present in environments where PAH pollutants are commonly encountered, *e.g.*, in seawater.^69^ Cellular necrosis within living systems caused by ischemia and reperfusion injury, other types of trauma,^70^ as well as by prolonged exposure to toxic substances such as PAHs^71^ results in the release of weakly chelated pools of iron and copper ions as necrotic cells burst, emptying denatured proteins and other debris into the extracellular environment.

In contrast to hydroxyl radicals which are generated through a Type I superoxide anion radical intermediate, there was no evidence indicating that Type II singlet oxygen plays a major role in the formation of anthracene **2** photosensitized direct strand breaks. Replacing H_2_O with D_2_O did not result in statistically significant increases DNA photocleavage on our nondenaturing agarose gels (Table 1).

**Figure 8:**
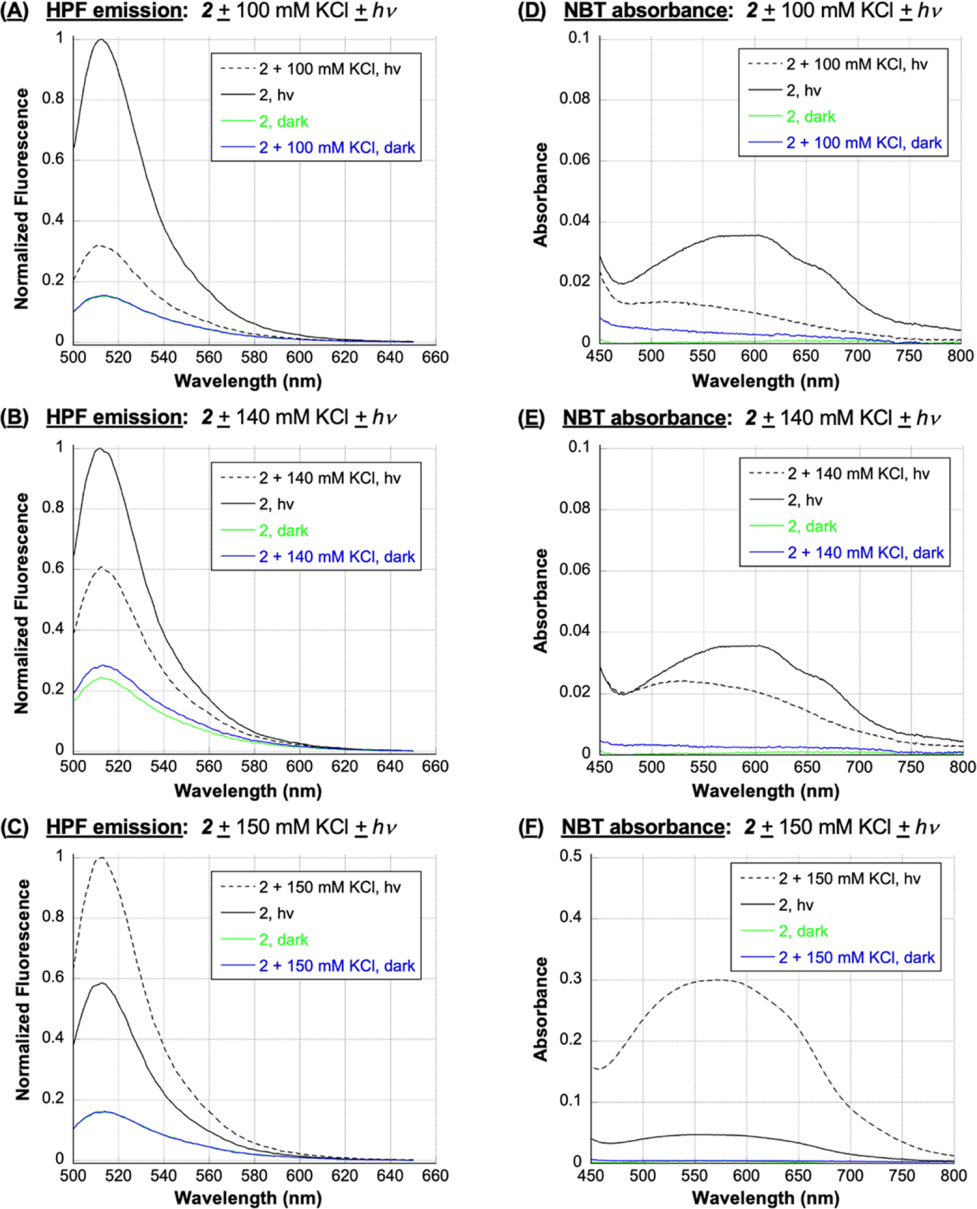
**HPF**: Representative, normalized HPF emission spectra of 3 μM HPF, 1 μM **2**, and 10 mM sodium phosphate buffer pH 7.0 in the presence and absence of (A) 100 mM KCl, (B) 140 mM KCl, and (C) 150 mM KCl (no DNA). Samples were irradiated for 7 min at 350 nm or kept in the dark. HPF emission spectra were then recorded from 500 to 650 nm at an excitation wavelength of 490 nm. **NBT**: Representative NBT absorption spectra of 16 μM NBT, 50 μM **2**, and 10 mM sodium phosphate buffer pH 7.0 in the presence and absence of (D) 100 mM KCl, (E) 140 mM KCl, and (F) 150 mM KCl (no DNA). Samples were irradiated for 5 min at 350 nm or kept in the dark. NBT absorption spectra were then recorded from 450 to 800 nm.

**Figure 9:**
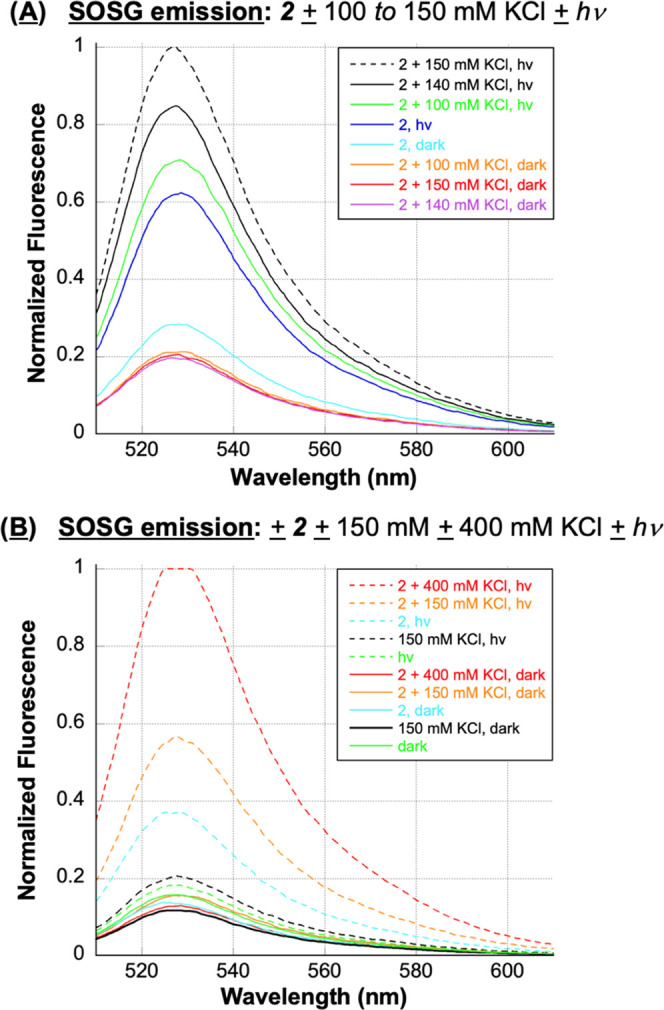
SOSG (A) SOSG emission spectra of 1 μM SOSG, 1 μM **2**, and 10 mM sodium phosphate buffer pH 7.0 in the presence and absence of 100, 140, and 150 mM KCl (no DNA). (B) Normalized SOSG emission spectra of 1 μM SOSG and 10 mM sodium phosphate buffer pH 7.0 in the presence and absence of 1 μM **2**, 150 mM KCl, and 400 mM KCl (no DNA). (A, B) Samples were irradiated for 30 min at 350 nm or kept in the dark. SOSG emission spectra were then recorded from 510 to 650 nm at an excitation wavelength of 500 nm.

In our next set of experiments, fluorometric and colorimetric probes were employed to confirm the involvement of key reactive oxygen species investigated in Table 1. We were also interested in examining the effects of different Cl^–^ concentrations on anthracene **2**-photosensitized ROS production. While chloride anions are present at high levels in seawater (∼500 mM),^40^ lower amounts of this anion are found in living systems. For example, extracellular chloride is ∼110 mM^72^ and within the cell cytoplasm and organelles, Cl^–^ ranges from 5 to 130 mM.^39^ The nuclear concentration of chloride anions in rat hepatocytes has been estimated to be ∼158 mM^34,37^ and in frog oocyte nuclei, 91.3 mM ± 9.0 with values ranging from 48.4 mM 6.5 to 172.6 mM ± 13 having been reported.^38^ Under similar ionic strength conditions, we then employed the colorimetric probe nitro blue tetrazolium chloride (NBT) and HPF to respectively test for superoxide anion radicals (O_2_^•–^) and hydroxyl radicals (^•^OH), and the fluorometric probe Singlet Oxygen Sensor Green (SOSG) to identify ^1^O_2_.^42^ In separate reactions, anthracene **2** and one of the three probes were irradiated at 350 nm in the absence and presence of 100, 140, and 150 mM concentrations of KCl (22 °C, pH 7.0; Figures 8 and 9A). (Since nucleic acids scavenge hydroxyl radicals and singlet oxygen, DNA was excluded from these experiments.) As expected, dark reactions and irradiated control samples containing probe and KCl in the absence of anthracene failed to generate significant fluorescence/absorption signals (Figures 8, 9A, and S6 in the Supporting Information). In contrast, the data obtained with irradiated *N*^1^*-*(anthracen-9-ylmethyl)ethane-1,2-diaminium dichloride **2** reactions revealed interesting trends. We will discuss the NBT and HPF data first (Figure 8). Scheme 2 proposes that superoxide anion radicals react to form hydroxyl radicals through a hydrogen peroxide intermediate. Accordingly, under no-salt conditions, both the colorimetric O_2_^•–^ probe NBT and the fluorometric ^•^OH probe HPF generated signals that were much higher compared to reactions containing 100 of KCl, moderately higher relative to 140 mM reaction signals, but lower compared to the signal intensities obtained using 150 mM KCl. In addition to supporting the mechanism in Scheme 2, Figure 8 suggests that a 150 mM concentration of KCl is very close to the lower cutoff limit for observing a Cl^–^-induced enhancement in *N*^1^*-*(anthracen-9-ylmethyl)ethane-1,2-diaminium dichloride **2** photosensitized hydroxyl radical production.

The data obtained from the fluorometric ^1^O_2_ probe SOSG was unexpected (Figure 9). In contrast to the DNA photocleavage chemical additive experiments in Table 1, which employed 400 mM KCl, there was clear evidence of a chloride salt-induced increase in levels of anthracene **2** photosensitized ^1^O_2_ over no-salt control reactions in the order 150 mM KCl > 140 mM KCl > 100 mM KCl > no-salt (Figure 9A). To reconcile these results with the 400 mM KCl Table 1 data, we conducted a follow-up experiment in the absence and presence of 150 mM and 400 mM KCl, and included dark reactions as well as irradiated controls containing probe in the absence of anthracene (Figure 9B). The resulting anthracene **2** photosensitized ^1^O_2_ levels were in the order 400 mM KCl ≫ 150 mM KCl > no-salt, with the signal generated at 400 mM KCl saturating the fluorescence detector. We concluded that chloride anions enhance the formation of anthracene **2**-photosensitized Type II singlet oxygen in addition to hydroxyl radicals. Singlet oxygen primarily generates minimally labile 8-hydroxy-2′-deoxyguanosine lesions and secondary ^1^O_2_ oxidation products that require hot piperidine treatment to generate DNA strand breaks,^60^ which may explain why singlet oxygen formation was not detected by our current and previously published^30^ gel assays of anthracene **2** (Table 1). The nondenaturing agarose electrophoresis method employed reveals only DNA direct strand breaks and does not detect guanine oxidation products.

In a previous publication, we assayed the integrity of the HPF and SOSG fluorometric probes used in the present manuscript.^73^ First, we demonstrated that hydroxyl radicals generated by the Fenton reagent (ammonium iron(II) sulfate + H_2_O_2_) were easily detected by HPF and that adding the ^•^OH scavenger sodium benzoate to the reaction significantly reduced HPF emission (Figure S7A in the Supporting Information).^73^ In evaluating SOSG, probe fluorescence was observed only upon photoactivation of methylene blue, a dual Type II singlet oxygen and Type I superoxide anion radical sensitizer in neutral media, and not in reactions containing the hydroxyl radical generating Fenton reagent (Figure S7B).^73,74^ In the current work, the following additional control experiments were performed. In separate reactions, anthracene **2** and either HPF, NBT, or SOSG were irradiated at 350 nm in the absence and presence of 400 mM KCl and a chemical additive: either sodium benzoate to detect hydroxyl radicals, deferoxamine mesylate to chelate Fe(III),^75^ Tiron to scavenge superoxide anion radicals, or 90% (*v*/*v*) D_2_O to increase the lifetime of singlet oxygen (no DNA; 22 °C, pH 7.0; Figure S8). As expected, controls without anthracene and/or *hν* exposure were all associated with low levels of fluorescence/absorption. In contrast, the optical signals generated by HPF, NBT, and SOSG were higher when anthracene **2** was irradiated in the presence of KCl compared to no-salt conditions (Figure S8). When chemical additives were included in parallel reactions, irradiation of *N*^1^*-*(anthracen-9-ylmethyl)ethane-1,2-diaminium dichloride **2** in the presence and absence of KCl revealed that: sodium benzoate decreases HPF fluorescence emission, confirming that HPF detects hydroxyl radicals (Figure S8A); deferoxamine mesylate decreases HPF emission, suggesting that Fe(III) plays a role hydroxyl radical production (Figure S8B); and Tiron lowers NBT absorption, indicating that NBT detects superoxide anion radicals (Figure S8B). When taken together, these findings support our hypothesis that anthracene **2** photosensitizes the production of hydroxyl radicals according to the Type I mechanism shown in Scheme 2. Moreover, when H_2_O was replaced with 90% D_2_O (*v*/*v*) in SOSG reactions, probe fluorescence increased, substantiating that SOSG detects Type II singlet oxygen and that anthracene **2** photosensitizes its production (Figure S8D).

### 3.4 Do Anthracene Photoproducts Contribute to the Formation of Hydroxyl Radicals?

Contrary to our notion that chloride anions might contribute to anthracene-photosensitized ROS production by serving as anthracene excited state electron donors (D^–^ in Scheme 2), Rehm–Weller calculations show that the free energy change (Δ*G*) of electron transfer from Cl^–^ to excited state anthracene is positive over a range of pH values from 4.0 to 11.0, suggesting that Cl^•^ radicals cannot be produced by anthracene.^51^ To the best of our knowledge, electron transfer from chloride anions to the triplet excited state of anthracene has never been observed in aqueous solutions.^76^ An alternative is to consider the involvement of redox-active anthraquinones and other photomodification products of anthracene.^21,77^ Anthraquinone triplet states have considerably more favorable reduction potentials^78^ to remove one electron from chloride anions and generate chlorine radicals. In flash photolysis experiments, Loeff and co-workers recorded transient absorption of 9,10-anthraquinone-2-sulfonate anion radicals (AQS^•–^) and halide radicals (X_2_^•–^) formed in aqueous solutions through interactions between triplet state ^3^ASQ* and high concentrations (> 0.5 M) of Cl^–^, Br^–^ and I^–^.^79^ (In the presence of halide anions, chlorine and other halogen radicals rapidly interconvert between monoatomic and diatomic forms: X^•^ + X^–^**⇆** X_2_^•–^.^80^) Loeff and co-workers also found that among the halides, Cl^–^ was the most effective in reducing 9,10-anthraquinone-2-sulfonate to AQS^•–^,^79^ which like anthracene, produces hydroxyl radicals according to Scheme 2.^81^ In a similar study by Kuzmin and Chibisov, flash photolysis of 9,10-anthraquinone-2, 6-disulfonic acid in the presence of 0.1 M Cl^–^ in pH 6.5 aqueous solutions gave rise to transient absorption consistent with the formation of a chlorine radical (Cl_2_^•–^) via the transfer of an electron from Cl^–^ to the anthraquinone excited triplet state.^82^ In the case of anthraquinones, it is therefore conceivable that the oxidation of chloride anions to chlorine radicals initiates the formation of hydroxyl radicals as shown in Scheme 2.

**Scheme 3:**
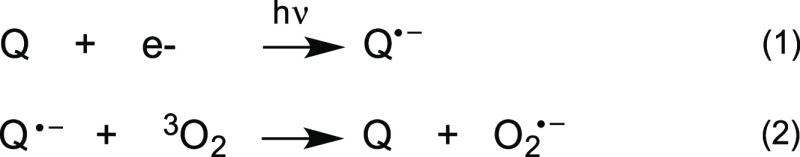
Reduction of a Quinone (Q) to a Semiquinone Anion Radical, Quinone Regeneration and Superoxide Anion Radical Release^77,83^

Irrespective of whether or not chloride anions serve as electron donors, the one-electron reduction of PAH quinones (Q) generates semiquinone anion radical intermediates (Q^•–^) that rapidly react with ground states triplet oxygen (^3^O_2_) to give rise to significant amounts of superoxide (O_2_^•–^) through the redox cycling pathway shown in Scheme 3.^77,83^ In this way, anthraquinone proto-products commonly formed upon the reaction of anthracene with either hydroxyl radicals^84^ or singlet oxygen easily generate superoxide (O_2_^•–^) that reacts further to produce hydroxyl radicals via steps 3 and 4 in Scheme 2. As a result, anthraquinone proto-products can display much higher toxicities compared to their parent anthracenes.^18,19,21^ This being said, we considered the possibility that photooxidation of *N*^1^*-*(anthracen-9-ylmethyl)ethane-1,2-diaminium dichloride **2** to one or more redox-active photoproducts might contribute to the high-level DNA-damaging hydroxyl radicals (and possibly chloride radicals) photosensitized by anthracene under high chloride salt conditions (Figures 1, 2, 4, 5, 6, 8, S5, S6, and S8).

To gain insight into the above hypothesis, we performed the following two experiments. In the first, we utilized anthraquinone-2-sulfonate (ASQ) (Figure S1), which, similar to anthracene,^52^ photosensitizes hydroxyl radical production through the participation of an initial electron donor atom (D^–^) followed by the formation of a superoxide anion radical intermediate (O_2_^•–^ in Scheme 2).^81,85^ As shown in Figure S9, upon irradiation with 350 nm UV light in the absence of KCl, the HPF signal was significantly higher for 0.5 μM anthraquinone-2-sulfonic acid compared to 1 μM anthracene **2**. Figure S9 additionally illustrates that KCl enhances anthracene-sensitized ROS production to a greater degree in the case of the anthraquinone. These findings led us to consider the possibility that, under high salt conditions, one or more oxidized photomodification products of **2** might contribute to ROS production.

**Figure 10:**
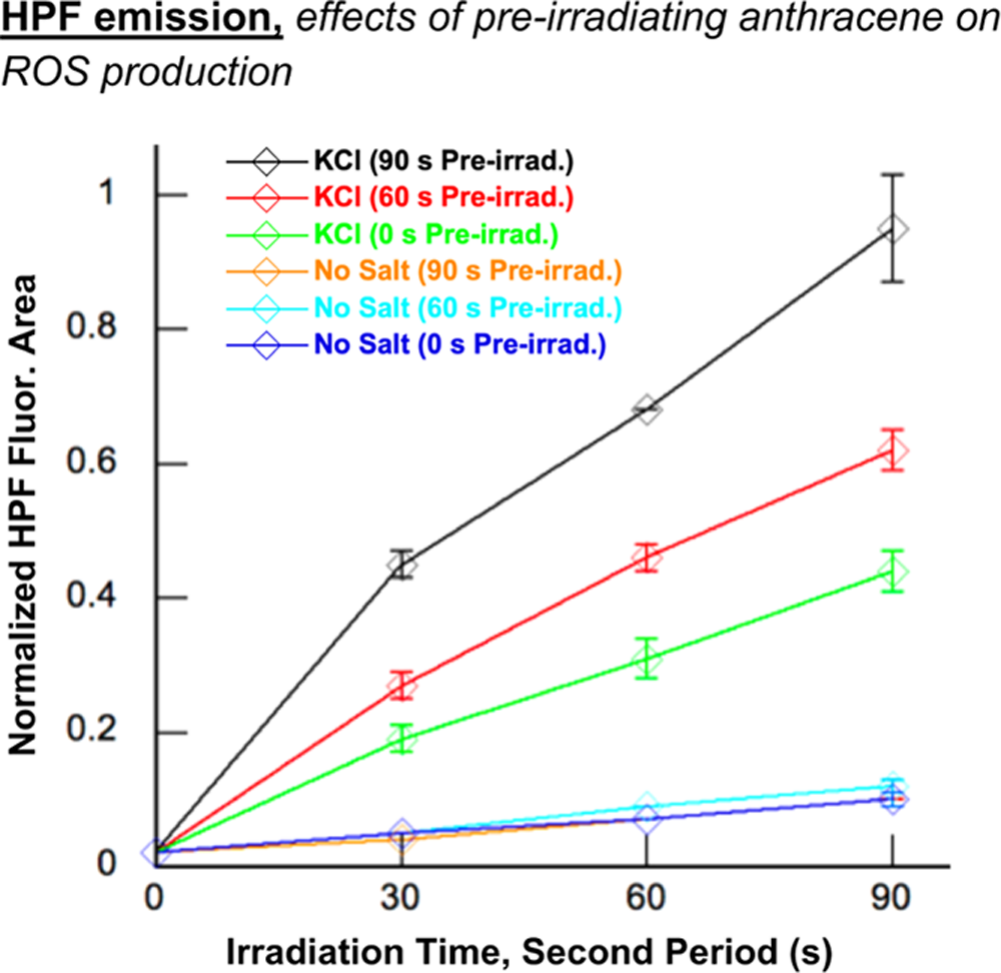
Stock solutions of 50 μM *N*^1^*-*(anthracen-9-ylmethyl)ethane-1,2-diaminium dichloride **2** in 10 mM sodium phosphate buffer pH 7.0 were pre-irradiated in a Rayonet photochemical reactor fitted with 8 24 W 350 nm UV lamps for 0, 60, and 90 s time intervals. Final concentrations of 400 mM KCl and/or 3 μM HPF were then added to the pre-irradiated anthracene solutions (1 μM final anthracene concentration; 10 mM sodium phosphate buffer pH 7.0). The new samples containing HPF and pre-irradiated anthracene were irradiated at 350 nm for 0, 30, 60, and 90 s. HPF fluorescence emission spectra were then recorded at an excitation wavelength of 490 nm. The graph shown above was generated by measuring the area underneath HPF fluorescence emission spectra from 500 to 650 nm over 2 to 3 reaction trials and then plotting the averaged area as a function of irradiation time. Error bars indicate standard deviation.

In our second experiment, we looked for evidence suggestive of the formation of anthracene photoproducts by pre-irradiating pH 7.0 buffered stock solutions of 50 μM anthracene **2** for 0, 60, and 90 s time intervals (350 nm). The stock solutions were then diluted to a 1 μM final anthracene concentration in the presence and absence of 400 mM KCl. After HPF fluorophore was added, the samples were irradiated at 350 nm for a second time, and HPF emission spectra were recorded after 0, 60, or 90 s. The area from 490 to 550 nm under each emission curve was then plotted as a function of the second irradiation time intervals (Figure 10). This was done to compare the effects of pre-irradiation on subsequent yields of anthracene-photosensitized ROS. The results show that, for the pre-irradiated anthracene **2** stock solutions, ROS production was higher when the KCl salt was present. The longer the pre-irradiation time, the more hydroxyl radicals were detected by HPF. In contrast, pre-irradiation had no effect on ROS levels when KCl was absent. These results suggest that one or more redox-active photoproducts of *N*^1^*-*(anthracen-9-ylmethyl)ethane-1,2-diaminium dichloride **2** might have been formed during pre-irradiation. The excited state(s) of this (these) putative photoproduct(s) might possess a more favorable reduction potential, as does anthraquinone, to participate in electron transfer with the chloride anion of KCl (or other electron donors) to generate hydroxyl radicals through an intermediate excited state anion radical (^3^PS*^•–^; Scheme 2).

**Figure 11:**
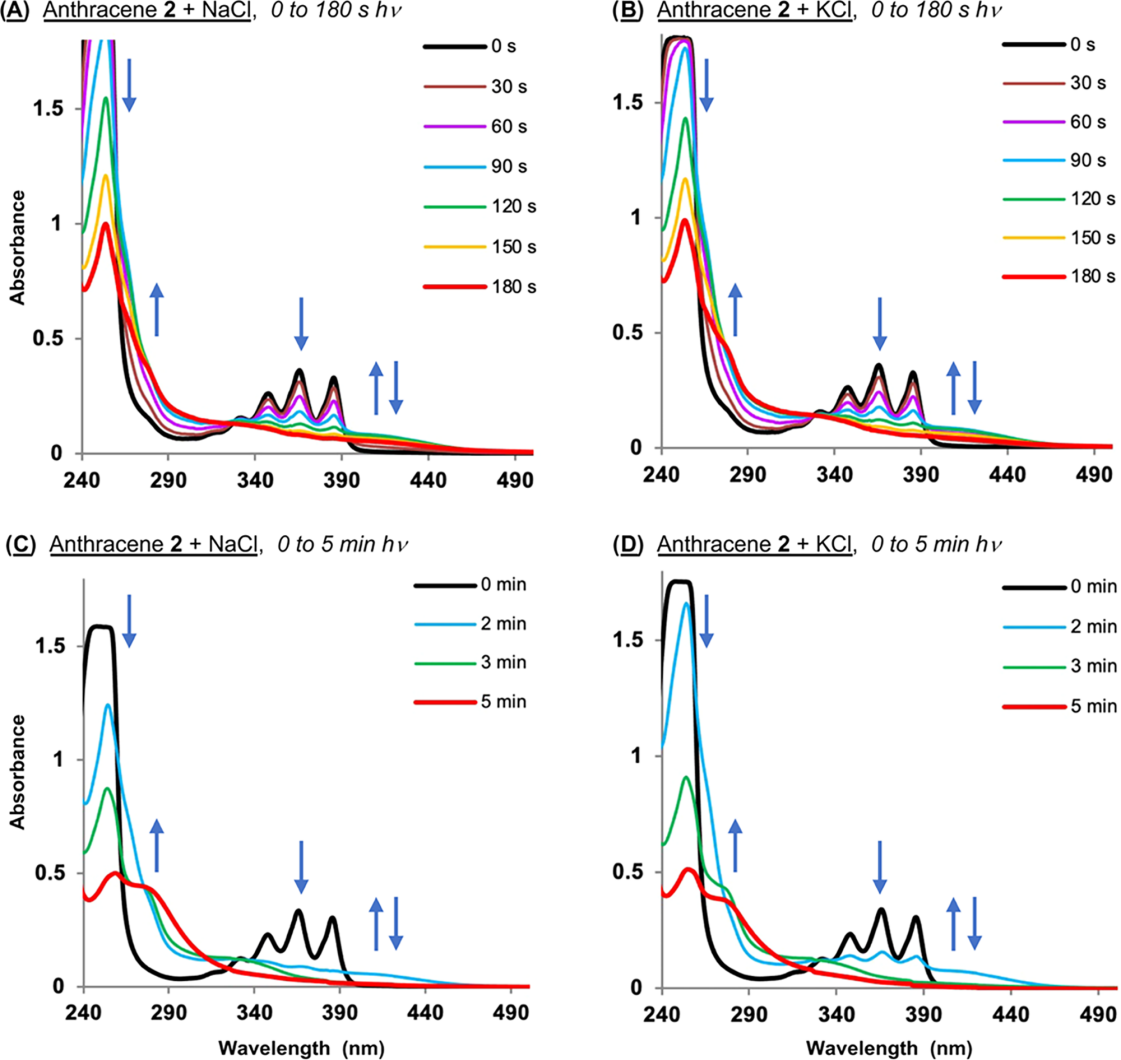
Representative spectra showing photoinduced degradation of 50 μM *N*^1^*-*(anthracen-9-ylmethyl)ethane-1,2-diaminium dichloride **2** in the presence and absence of 400 mM of chloride salts in 10 mM sodium phosphate buffer pH 7.0. Samples were irradiated at 350 nm for time intervals from: (A, B) 0 to 180 s and (C, D) 0 to 5 min in a ventilated Rayonet photochemical reactor fitted with 8 RPR-3500 Å, 24 W lamps.

Evidence of the formation of anthracene **2** photoproducts is present in the photodegradation studies shown in Figures S5 and 6, in which absorption spectra of **2** were recorded in the presence and absence of salt over irradiation time intervals ranging from 0 to 5 min (350 nm; no DNA). As discussed, we observed that ionic-strength-dependent rates of anthracene photodegradation occurred in the order KCl ≈ NaCl ≫> NaF ≈ KF > no-salt > KBr ≈ NaBr (Figures 5 and S5; iodide salt data could not be collected due to extreme sample turbidity). Shown in Figure 11 are the “*hν* = 0 to 180 s” anthracene **2** chloride salt UV–visible spectra taken from Figure S5 presented next to chloride salt spectra recorded over 0 to 5 min of irradiation at 350 nm. The data show that the halide salts promote: (i) the loss of anthracyl vibration fine structure; (ii) the formation of one (or more) blue-shifted species that absorb(s) light between ∼280 and 320 nm; and (iii) new ∼400 to 440 nm absorption that first emerges and, for NaCl and KCl, then dissipates at longer irradiation times.

In an attempt to identify anthracene **2** photomodification products that might account for the observed absorption changes, ESI-MS spectra were initially acquired for reactions containing 1 and 10 mM **2** in the presence and absence of 400 mM KCl (10 mM sodium phosphate buffer pH 7.0). Individual samples were irradiated at 350 nm for either 0, 2, or 10 min, but there were no significant differences between the mass spectra of irradiated reactions compared to those of corresponding dark controls (data not shown). In order to mitigate possible ion suppression effects that can confound the ESI analyses of complex mixtures,^86^ in our next set of experiments, we replaced the phosphate buffer with 10 mM sodium formate pH 7.0 and lowered the salt concentration from 400 to 150 mM KCl. Reactions containing 1 mM **2**, the minimum dye concentration needed to obtain an acceptable ESI single-to-noise ratio under the high-ionic-strength conditions employed, were then irradiated at 350 nm for either 0, 2, 5, or 60 min. Shown in Figures S11 and S12 are representative 0 and 60 min ESI-MS spectra. Peaks corresponding to the parent ion of **2** (*m*/*z* = 251.16) and its anthracen-9-yl methylium ESI-MS fragmentation product (*m*/*z* = 191.01) are present in all of the images acquired (Figures S11 and S12), whereas ESI peaks with *m*/*z* ratios of 459.24(5), 501.30, and 539.25(6) appear only in the light reactions and increase in intensity as a function of increasing irradiation time (Figures S11B and S12B). The ESI signal with an *m*/*z* ratio of 539.25(6) likely represents a K^+^ adduct of the 501.30 *m*/*z* species. While we have tentatively identified the 459.24(5) and 501.30 *m*/*z* peaks as possible ESI artifacts arising from the reaction of the *m*/*z* = 191.01 anthracen-9-yl methylium fragmentation product with anthracene **2** (Figures S11 and S12), it is very interesting that the ESI signals of these species are not observed in dark reactions in which the fragmentation product and the parent ion of **2** are simultaneously present (Figures S11A and S12A). We will establish in future studies the structures of the compounds that are generating the mysterious *m*/*z* = 459.24(5) and = 501.30 peaks. Unfortunately, none of the *m*/*z* ratios seen in our experiments are consistent with oxidation products that are known to form upon the reaction of anthracenes with hydroxyl radicals and singlet oxygen.^77^ One complicating factor could be the ease at which *N*^1^*-*(anthracen-9-ylmethyl)ethane-1,2-diaminium dichloride **2** releases its *m*/*z* = 191.01 anthracen-9-yl methylium fragmentation product, which is the dominant peak in the majority of ESI spectra we recorded (Figures S11 and S12). Oxidation products of **2** could be fragmenting in a similar fashion, complicating their accurate detection and identification. Using identical conditions, we then acquired the ESI mass spectrum (Figure S13) of commercially available 9-(methylaminomethyl)anthracene (Figure S1), a 9-(aminomethyl)anthracene similar in structure to **2**. Interestingly, the anthracen-9-yl methylium ESI-MS fragmentation product (*m*/*z* = 191.01) of the anthracene is the major peak in the spectrum while the parent ion of 9-(methylaminomethyl)anthracene (*m*/*z* = 222.128) is not observed (Figure S13).

### 3.5 DNA Interactions

Our results thus far show that both KCl and NaCl increase anthracene-photosensitized hydroxyl radical production in a similar fashion (Figures 4 and 5) while only KCl significantly enhances DNA direct strand break formation over no-salt controls (Figures 2 and 3). It is clear that the effects of potassium and sodium counter cations on anthracene/DNA interactions should be considered in order to attempt to explain this discrepancy. Toward this end, we recorded UV–visible spectra of compound **2** in the presence and absence of 200 μM bp of calf thymus (CT) DNA and 400 mM of the eight halide salts (10 mM sodium phosphate buffer; Figure S10). Under no-salt conditions, the addition of the CT DNA to **2** induces significant hypochromicity and large bathochromic shifts in the anthracene vibrational fine structure with respect to free dye, spectral changes that are hallmarks of anthracene intercalation (Figure S10A).^87^ When each one of the eight salts is added, moderate hypochromicity and minimal red-shifting of the anthracene vibronic peaks occur with respect to the free dye, spectral trends that are suggestive of a disruption of intercalative interactions under the no-salt conditions in favor of external and/or groove DNA binding modes.^30,31,87^ These salt-induced changes in binding could increase DNA damage by making anthracene more accessible to ^3^O_2_ and therefore, better able to photosensitize ROS production.^30,31,88^ However, the spectra containing salts are similar with respect to the intensity and the position of the anthracene peaks (Figure S10B through I), indicating that all eight Na^+^ and K^+^ halides have comparable effects on the DNA binding modes of anthracene **2**. Yet only KCl increases anthracene-sensitized DNA photocleavage to a significant degree (Figures 1 and 2).

**Figure 12:**
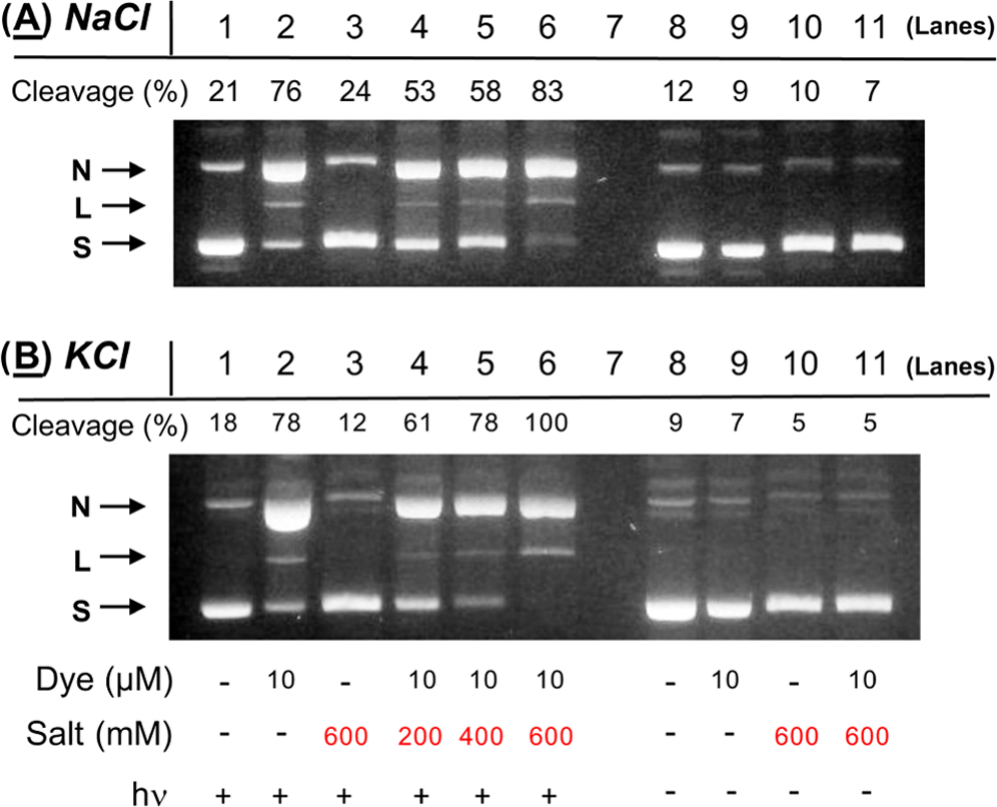
Photographs of 1.5% nondenaturing agarose gels showing photocleavage of 38 μM bp pUC19 plasmid DNA in the presence and absence of 10 μM (9-anthracenylmethyl)trimethylammonium chloride and 200 to 600 mM of: (A) NaCl and (B) KCl (10 mM sodium phosphate buffer pH 7.0). The reactions in Lanes 1 to 6 were irradiated for 60 min in a ventilated Rayonet photochemical reactor fitted with 8 RPR-3500 Å, 24 W lamps.

The ability of anthracene **2** (Scheme 1) to interact with alkali cations was considered next. For example, the ethylenediamine unit of **2** could form a five-membered chelate ring with either Na^+^ and/or K^+^. The change in ligand conformation accompanying complex formation with either cation could subtly alter DNA binding interactions and thereby contribute to the different levels of DNA photocleavage exhibited by **2** when NaCl vs KCl is present. In order to test this hypothesis, DNA photocleavage experiments were conducted utilizing the quaternary amine (9-anthracenylmethyl)trimethylammonium chloride (Figure S1 in the Supporting Information), a chromophore that is devoid of metal binding nitrogen donor atoms. DNA samples were irradiated at 350 nm in the presence of NaCl and KCl concentrations ranging from 0 to 600 mM and then electrophoresed. Figure 12 shows that DNA photocleavage was higher for KCl at each of the three halide salt concentrations tested. These results point to a mechanism in which metal-ligand interactions involving Na^+^ or K^+^ cations and the amine groups in the ethylenediamine unit of **2** are unlikely to play a major role in anthracene-sensitized DNA photocleavage.

To account for the ability of KCl to enhance anthracene **2** photosensitized DNA direct strand break formation to a significantly greater degree than NaCl, we next considered the differential effects of Na^+^ and K^+^ on DNA structure. Although much work has been done to gain a clear understanding of the interactions of Na^+^ and K^+^ cations with DNA, many questions remain unanswered. The counter cations are not clearly visible by NMR as well as X-ray diffraction, where it is challenging to distinguish Na^+^ and K^+^ from ordered water molecules in electron density maps.^89,90^ Based on counterion condensation theory,^91^ sodium(I) and potassium(I) cations have been predicted to form a nonspecific mobile ion atmosphere around duplex DNA.^90^ More recent studies have pointed to the existence of sequence-specific monovalent cation binding sites in the DNA grooves.^89,90^

Upon interacting with DNA, counter cations can trigger alterations in duplex DNA structure.^89,90^ While some theoretical studies report that these structural changes are independent of the nature of the counter-cation, others point to significant differences between Na^+^ and K^+^.^90^ In experimental work examining duplex DNA in solution, NMR data show that Na^+^ and K^+^ differentially alter duplex DNA backbone torsional angles in a sequence-dependent fashion.^89^ Electrophoretic mobility measurements of AT-rich sequences recorded by Feigon and co-workers demonstrate that potassium(I) increases global bending to a greater degree than sodium(I).^92^ Additionally, Na^+^ and, to a greater extent, K^+^ promote the formation of G-quadruplex structures in double-helical DNA, while Cs^+^ causes G-quadruplex unfolding.^93^ In broader studies, circular dichroism in combination with electrophoretic mobility data reveal that monovalent cations increase helical twist angle in the order Na^+^ < K^+^ < Cs^+^ < NH_4_^+^, causing DNA to overwind and vertical rise between base pairs to decrease in a commensurate fashion.^94^ This being said, we hypothesized that subtle structural changes afforded by KCl might make DNA more susceptible than NaCl to degradation by ROS in anthracene **2** photocleavage reactions. DNA direct strand breaks occur when hydroxyl radicals abstract hydrogen atoms from 2-deoxyribose.^7^ If potassium(I) cations were to make the 2-deoxyribose hydrogen atoms more susceptible to radical attack, then a higher degree of DNA photocleavage would be expected. The ability of Na^+^ and K^+^ cations to encourage a transition from anthracene intercalation to oxygen-accessible minor groove and external DNA binding modes could also be an important factor (Figure S10).^30,31,87,88^

**Figure 13:**
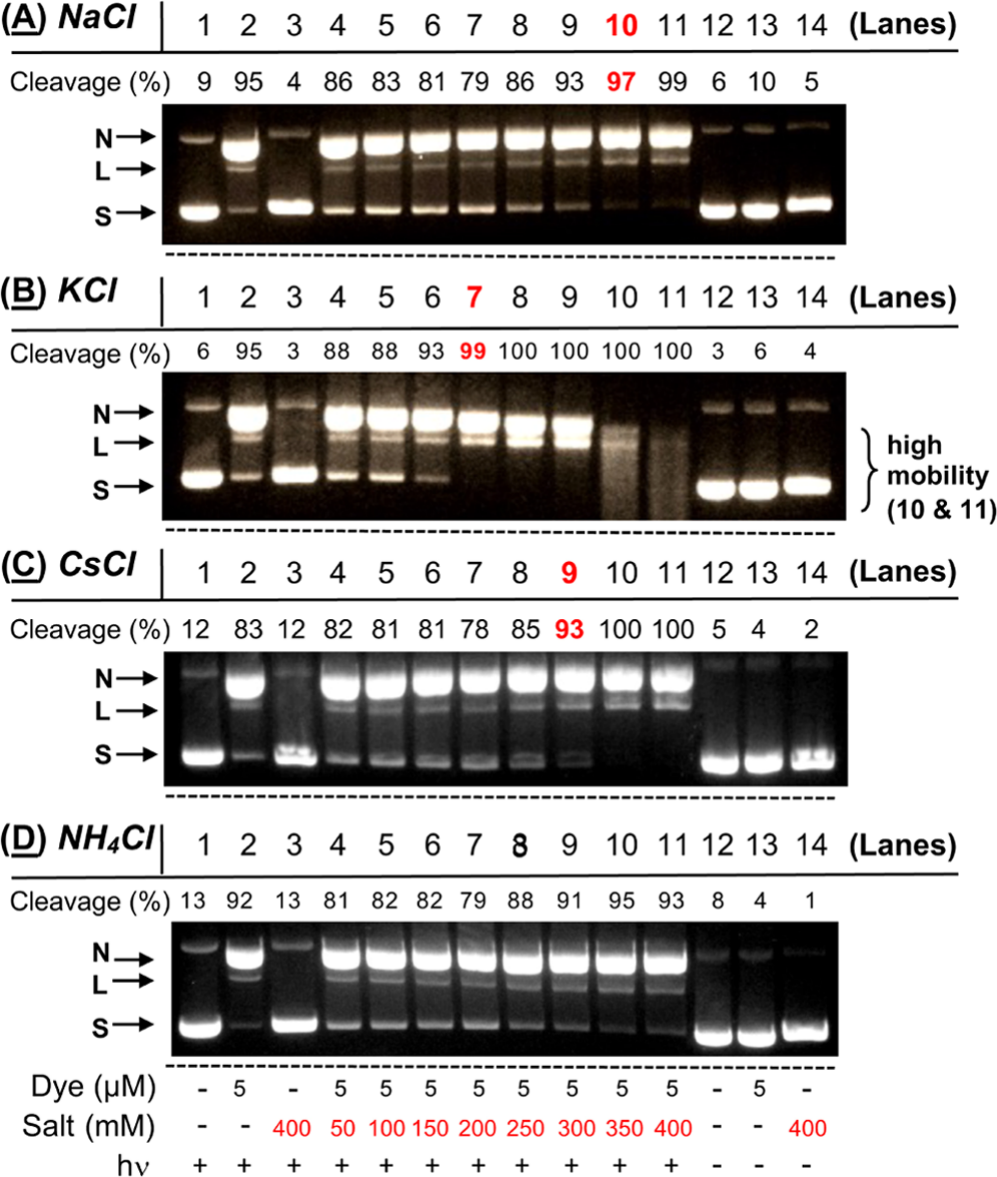
Photographs of 1.5% nondenaturing agarose gels showing photocleavage of 38 μM bp pUC19 plasmid DNA in the presence and absence of 5 μM *N*^1^*-*(anthracen-9-ylmethyl)ethane-1,2-diaminium dichloride **2** and 50 to 400 mM of: (A) NaCl, (B) KCl, (C) CsCl, and (D) NH_4_Cl (10 mM sodium phosphate buffer pH 7.0). The reactions in Lanes 1 to 11 were irradiated for 60 min in a ventilated Rayonet photochemical reactor fitted with 8 RPR-3500 Å, 24 W lamps. (Panels A and B also appear in Figure 2 of this manuscript.) Highlighted in red are the first reactions in each series in which there is a statically significant salt-induced photocleavage enhancement relative to the no-salt control reactions in Lane 2.

As an indirect test for cation-induced effects on DNA structure and interactions, we compared cesium and ammonium chloride salts to NaCl and KCl in photocleavage reactions (Figures 2 and 13). Solutions containing pUC19 plasmid DNA and *N*^1^*-*(anthracen-9-ylmethyl)ethane-1,2-diaminium dichloride **2** were irradiated at 350 in the presence of an increasing concentration of chloride salt ranging from 50 mM up to 400 mM (pH 7.0, 60 min, 25 °C). The ionic radii for Na^+^, K^+^, Cs^+^, and NH_4_^+^ are 116, 152, 181, and 175 pm, respectively,^95^ and as mentioned above, the cations increase DNA helical twist angle in the order Na^+^ < K^+^ < Cs^+^ < NH_4_^+^.^94^ The comparison in Figure 13 shows that raising the salt concentration increased photocleavage yields in all cases, but that the series of chloride salts enhanced DNA photocleavage over no-salt conditions in the order: KCl ≫> CsCl > NaCl > NH_4_^+^. These results suggest that quadruplex DNA does not play a major role in DNA photocleavage, especially taking into consideration that there are no strong G-quartette forming sequences in native pUC19 plasmid.^96^ There is also no direct correlation between the ionic radius and the degree of helical overwinding exhibited by the different counter cations with corresponding DNA photocleavage yields. Notwithstanding, overwinding and other salt-induced changes in DNA structure might still be important in enabling K^+^ to dramatically increase anthracene-photosensitized DNA damage over Na^+^ and the other counter cations we tested. The precise amount of overwinding afforded by K^+^ might be optimal for DNA photocleavage, for example. In addition to subtle, cation-specific changes in DNA structure, there may be additional, unknown variables that account for the particularly high activity exhibited by KCl toward anthracene **2**-sensitized DNA photooxidation. Notwithstanding, when combined with a favorable chloride anion-induced heavy-atom effect, the increased accessibility to ground-state triplet oxygen afforded by a salt-induced change in DNA binding mode from intercalation to groove binding and external interactions is likely to contribute to the high levels of hydroxyl radicals and singlet oxygen photosensitized by **2** when chloride anions are present.

## 4 Conclusions

In this work, we have compared the effects of 400 mM NaCl and KCl on *N*^1^*-*(anthracen-9-ylmethyl)ethane-1,2-diaminium dichloride **2** photosensitized DNA cleavage and ROS production to their counterpart Na^+^ and K^+^ fluoride, bromide, and iodide salts. In a fluorescence assay using HPF to detect hydroxyl radicals (no DNA), only NaCl and KCl were capable of significantly enhancing ROS production over no-salt controls (Figure 4). UV–visible spectra showed that, in the presence of the eight salts, anthracene **2** is stable over time and absorbs similar amounts of light (Figures S4 and S10). Therefore, salt-induced changes in anthracene absorption can be ruled out as a major contributing factor. Halides have been shown to impact anthracene triplet state yields and lifetimes by affecting rates of intersystem crossing.^50^ In the presence of the eight halide salts, anthracene **2** fluorescence decreases in the order F^–^ > Cl^–^ > Br^–^ > I^–^ (Figure 7), pointing to a possible heavy-atom effect.^50,51^ In frozen aqueous micellar solutions, iodide is associated with the shortest lifetime and highest yield, followed by bromide, chloride, and fluoride.^50^ Conversely, fluoride has the longest anthracene triplet state lifetime and lowest yield, followed by chloride, bromide, and iodide.^50^ In other words, despite iodide showing the highest triplet state yield, the lifetime of the anthracene triplet state is too short for an interaction with ground-state triplet oxygen to take place. In the case of fluorides, the triplet state yield may be too low to give rise to significant amounts of ROS. Among the four halide anions, chloride may afford the optimum balance between triplet state yield and the lifetime needed to increase the levels of anthracene-photosensitized ROS.

In order to investigate both Type I and Type II reaction mechanisms, plasmid DNA photocleavage reactions that contained the chemical additives EDTA, Tiron, catalase, and sodium benzoate in the absence and presence of 400 mM KCl were resolved by agarose gel electrophoresis, a method that detects DNA direct strand breaks but does not reveal guanine oxidation products generated by singlet oxygen. The results suggested that, under high-ionic-strength conditions, metal ions, superoxide anion radicals, hydrogen peroxide, and hydroxyl radicals contribute to the formation of anthracene **2** photosensitized direct DNA strand breaks (Table 1) through the Type I electron transfer mechanism shown in Scheme 2. Type II singlet oxygen formation could not be inferred when D_2_O was employed, however. Compared to hydroxyl radicals, which efficiently cleave DNA, singlet oxygen may only be capable of indirectly contributing to the formation of low levels of direct strand breaks.^31,61−65^ Notwithstanding, subsequent reactions containing the hydroxyl radical probe HPF and Singlet Oxygen Sensor Green showed that KCl concentrations ranging from 150 to 400 mM and 100 to 400 mM, respectively, enhanced *N*^1^*-*(anthracen-9-ylmethyl)ethane-1,2-diaminium dichloride photosensitized ^•^OH and ^1^O_2_ production over no-salt controls (no DNA; Figures 4, 8, 9, and S6). Taken together, the data lead us to conclude that chloride anions increase the formation of both anthracene **2** photosensitized Type II singlet oxygen in addition to hydroxyl radicals (Scheme 2) over a wide range of salt concentrations.

When the eight halide salts were compared side by side in DNA photocleavage experiments, only KCl and, to a much lesser extent NaCl, enhanced anthracene **2**-sensitized photocleavage over no-salt control reactions (Figure 1–3). The UV–visible spectra presented in this report, however, show that the eight sodium(I) and potassium(I) halides induce similar changes in DNA binding interactions (Figure S10). In all cases, the salts appear to induce an alteration in anthracene **2** binding mode from intercalation to groove and/or external interactions in which the chromophore would be more exposed to the ground-state triplet oxygen molecules and, in theory, better able to generate ROS.^30,31,88^ Yet, only KCl is capable of inflicting major amounts of DNA direct strand breaks compared to no-salt controls (Figures 1–3). Our cumulative findings underscore the importance of an optimal heavy-atom effect exhibited by chloride anions on anthracene triplet state lifetimes and yields combined with favorable salt-induced changes in anthracene/DNA binding mode. When DNA is absent, both NaCl and KCl increase levels of anthracene-photosensitized hydroxyl radical production in a similar fashion, while the fluoride, bromide, and iodide salts are all associated with lower ROS levels (Figures 4 and 5). When DNA is present, it cannot be ruled out that a subtle potassium(I)-induced alteration in DNA structure makes 2-deoxyribose hydrogen atoms more susceptible to abstraction by hydroxyl radicals compared to sodium(I), accounting for the unique ability of KCl to enhance DNA photocleavage. Our results also suggest that photooxidation of 9-aminomethylanthracenes in aqueous environments may produce oxidized anthracene products that react further with UV light to give rise to additional DNA-damaging hydroxyl radicals (Figures 6, 10, and S5).

The concentrations of Na^+^ and K^+^ cations in the cell nucleus are relatively high, ∼150 and ∼260 mM, respectively.^32−34,36^ Moreover, Cl^–^ concentrations are elevated in seawater (∼500 mM)^40^ and range from 5 to 130 mM in the cell cytoplasm and organelles^39^ with nuclear concentrations reported to be as high as 91.3 mM ± 9.0 up to 158 mM^34,37,38^ Thus, our findings may be relevant to the phototoxicity of anthracenes in different environments with high chloride levels. There are reports on the increased salinity of fresh waters due to human activities such as de-icer use in airports and roadways.^97^ Given the ubiquitous presence of anthracenes and other PAHs in aquatic milieus, chloride salts may amplify anthracene phototoxicity in marine microorganisms and plants. Moreover, the phototoxicity of dietary supplements containing anthraquinones may also be influenced by sodium(I) and potassium(I) cations as well as by chloride anions in living organisms.

## Supporting Information Available

Anthracene structures (Figure S1); DNA photocleavage, dark controls (Figure S2); HPF fluorescence emission spectra with halide salts (no **2**) (Figure S3); UV–vis absorbance spectra of **2***vs* time (Figure S4); UV–vis absorbance spectra of **2***vs* 350 nm hν (Figure S5); HPF and NBT detection of ROS photosensitized by **2**, multiple trials with controls (Figure S6); HPF and SOSG detection of ROS generated by the Fenton reagent (dark) and methylene blue (hν) (Figure S7); HPF, NBT, and SOSG detection of ROS photosensitized by **2**, with and without chemical additives (Figure S8); HPF fluorescence emission spectra of 9,10-anthraquinone-2-sulfonate vs **2** (Figure S9); UV–vis absorbance spectra of **2** with and without DNA/halide salts (Figure S10); ESI mass spectra of **2** (Figures S11 and S12); ESI-MS spectrum of 9-(methylaminomethyl)anthracene (Figure S13); and analytical data and ^1^H- and ^13^C- NMR spectra of **2** (Figures S14–S16) (PDF)

## Dedication

^†^ This manuscript is dedicated to Elysse M. Craddock, Professor Emerita, in celebration of her mentorship to students and outstanding contributions to the field of developmental biology.
