# Supplementary material for: New Insights into the Phototoxicity of Anthracene-Based Chromophores: The Chloride Salt Effect†
Source: Chem Res Toxicol. 2023 Jun 22;36(7):1002–20. doi: 10.1021/acs.chemrestox.2c00235 (PMC10354805; doi:10.1021/acs.chemrestox.2c00235)
Supplement: Supplementary file 1 — tx2c00235_si_001.pdf [file tx2c00235_si_001.pdf]

Supporting Information

New Insights into the Phototoxicity of Anthracene-based Chromophores: The Chloride Salt Effect

Mohammad Sadegh Safiarian,[#] Aikohi Ugboya,[#] Imran Khan, Kostiantyn O. Marichev, and Kathryn B. Grant*

Department of Chemistry, Georgia State University, Atlanta, GA 30303 USA

[#]These authors contributed equally to this work.

Table of Contents: **Page**

Figure S1: Structures of anthracene, hypericin, *aloe emodin*, anthraquinone -2-sulfonate, N^1-(anthracen-9-ylmethyl)ethane-1,2-diaminium dichloride 2, 9-(methylaminomethyl)anthracene.______ **S2**

Figure S2: Photocleavage of plasmid DNA in the presence of **2** and halide salts with dark controls.______ **S3**

Figure S3: Fluorescence emission spectra of HPF in the presence of 400 mM of halide salts (no **2**).______ **S4**

Figure S4: UV-vis absorbance of **2** in the presence and absence of halide salts *vs* time.___ **S5**

Figure S5: Photo-induced degradation of N^1-(anthracen-9-ylmethyl)ethane-1,2-diaminium dichloride **2** in the presence and absence of halide salts *vs* time.______ **S6**

Figure S6: HPF and NBT detection of ROS photosensitized by **2**, multiple trials with controls.______ **S7**

Figure S7: HPF and SOSG detection of ROS generated by the Fenton reagent (dark) and methylene blue (638 nm hν).______ **S8**

Figure S8: Effects of chemical additives on HPF, NBT, and SOSG detection of ROS photosensitized by **2**, multiple trials with controls.______ **S9**

Figure S9: HPF fluorescence emission spectra after 350 nm irradiation of 9,10-anthraquinone-2-sulfonate *vs* **2**.______ **S10**

Figure S10: UV-vis abs. spectra of **2** in the presence and absence of DNA, halide salt.____**S11**

Figure S11: ESI-MS spectra of 1 mM of **2**, $\pm$ hν, no-salt.____________________________ **S12**

Figure S12: ESI-MS spectra of 1 mM of **2**, $\pm$ hν, 150 mM KCl.______________________ **S13**

Figure S13: ESI-MS spectrum of 1 mM of 9-(methylaminomethyl)anthracene.__________ **S14**

Figure S14: Anal. data of N^1-(anthracen-9-ylmethyl)ethane-1,2-diaminium dichloride **2**.__ **S15**

Figure S15: ^{1}H-NMR spectrum of **2**.____ **S15**

Figure S16: ^{13}C-NMR spectrum of **2**.____ **S16**

Reference.____ **S16**

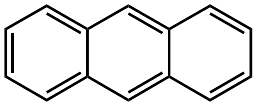

anthracene

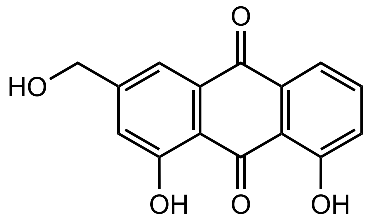

aloe-emodin

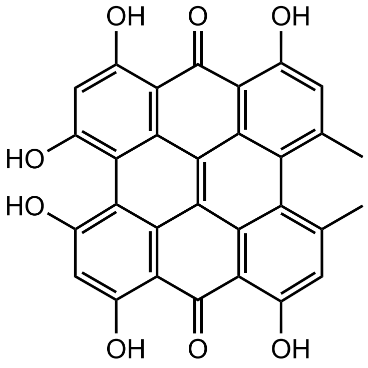

hypericin

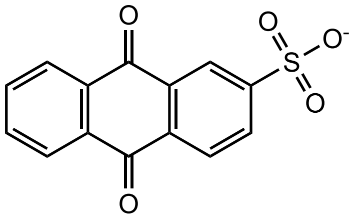

9,10-anthraquinone-2-sulfonate

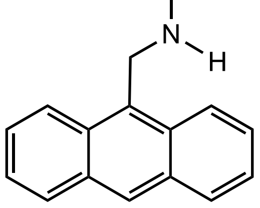

9-(methylaminomethyl)
anthracene

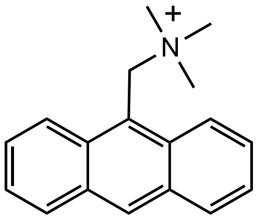

(9-anthracenylmethyl)
trimethylammonium chloride

Figure S1. Structures of anthracene, hypericin, *aloe-emodin*, anthraquinone-2-sulfonate, 9-(methylaminomethyl)anthracene, and (9-anthracenylmethyl)trimethylammonium chloride.

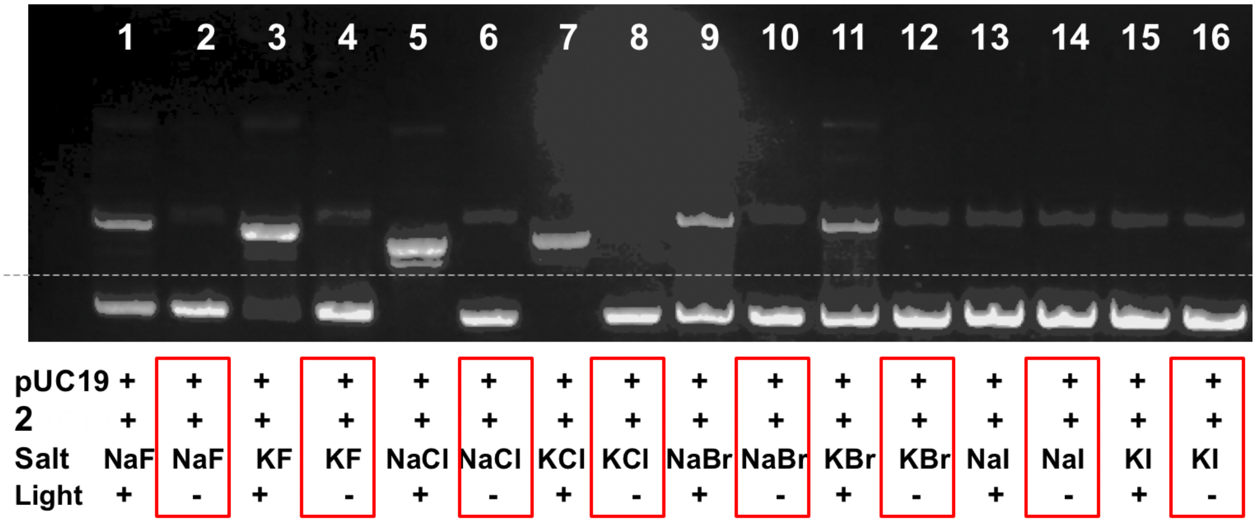

Figure S2. Photograph of a 1.5% non-denaturing agarose gel showing photocleavage of 38 μM bp of pUC19 plasmid DNA in the presence and absence of 1 μM of N^1-(anthracen-9-ylmethyl)ethane-1,2-diaminium dichloride **2** and 410 mM of NaF, KF, NaCl, KCl NaBr, KBr, NaI, or KI. Samples contained 10 mM sodium phosphate buffer pH 7.0 and 38 μM bp of the DNA. The reactions in Lanes 1, 3, 5, 7, 9, 11, 13, and 15 were irradiated at 350 nm for 60 min (25 ˚C). The lanes boxed in red were kept in the dark (60 min, 25 ˚C).

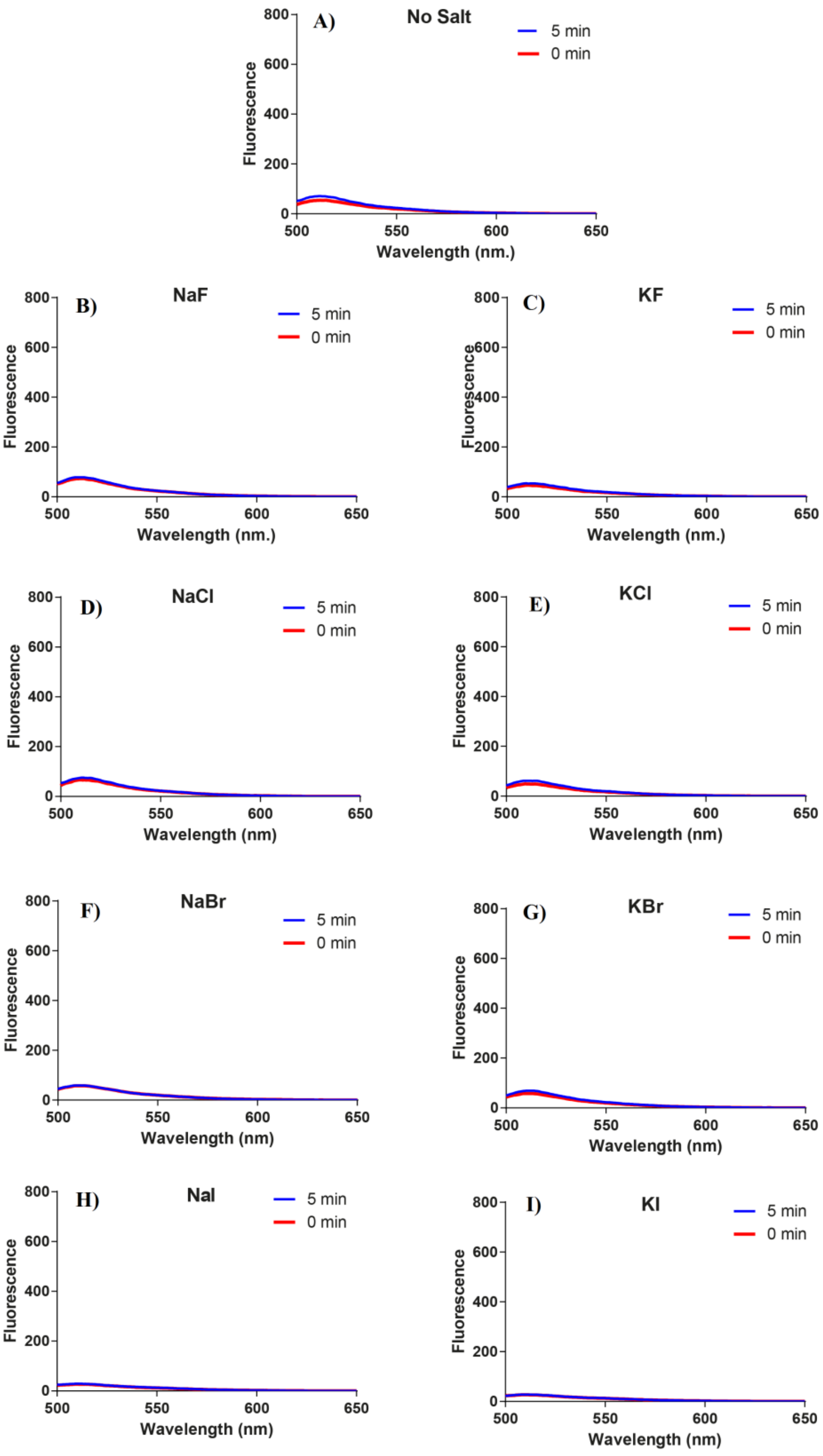

Figure S3 Fluorescence emission spectra of 3 µM HPF **without** anthracene **2** in the **A**) absence and presence of 400 mM of **B**) NaF, **C**) KF, **D**) NaCl, **E**) KCl, **F**) NaBr, **G**) KBr, **H**) NaI and **I**) KI (10 mM sodium phosphate buffer pH 7.0). Samples were irradiated for 5 or 0 min in a ventilated Rayonet photochemical reactor with 8 RPR-3500 Å, 24 W lamps. HPF fluorescence emission spectra were recorded from 500 to 650 nm using an excitation wavelength of 490 nm.

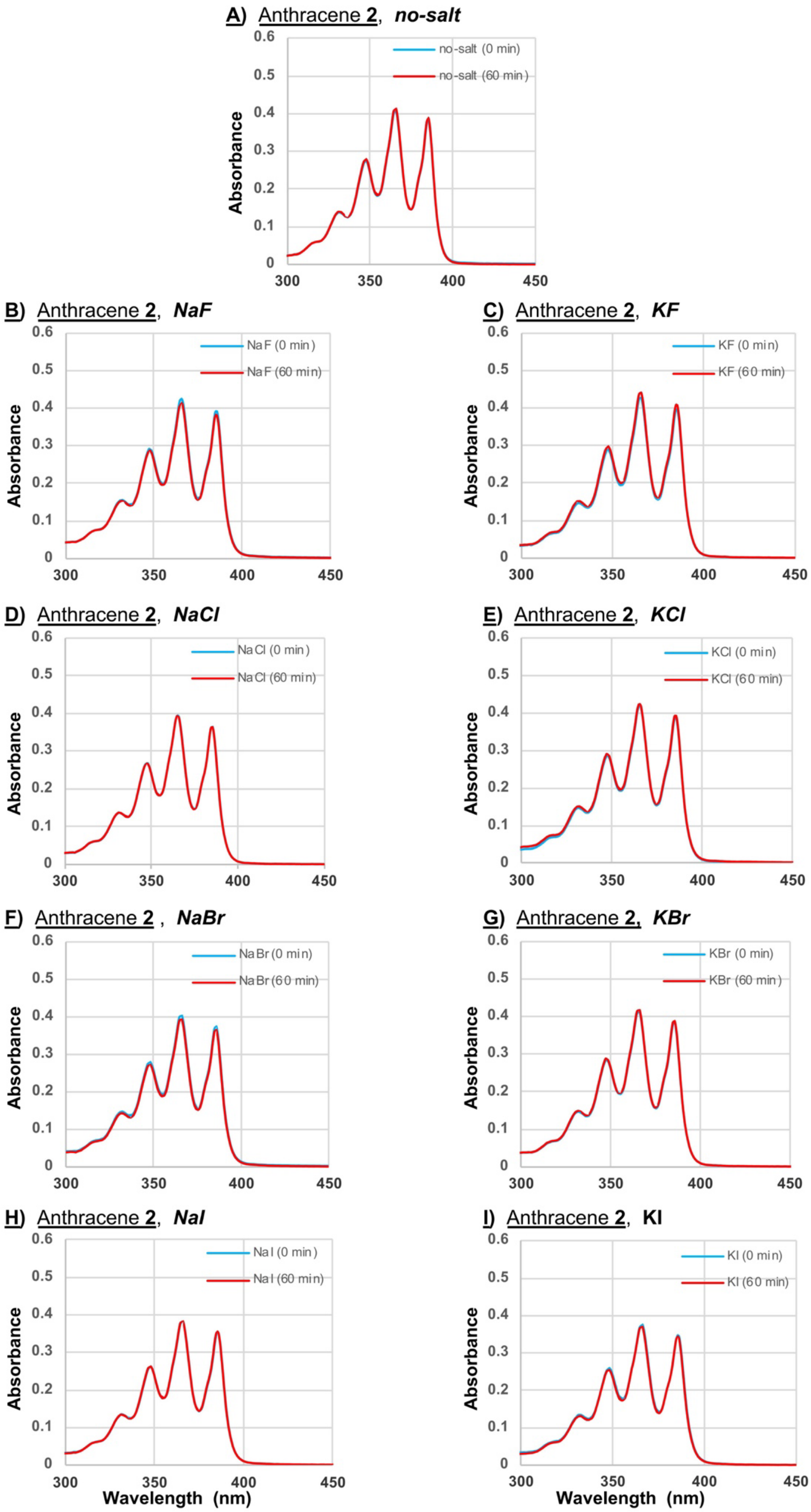

Figure S4. UV-visible absorbance of 50 µM of *N*[1]-(anthracen-9-ylmethyl)ethane-1,2-diaminium dichloride **2** in the presence of 400 mM of NaF, KF, NaCl, KCl, NaBr, KBr, NaI, and KI at 0 and 60 min time points. All samples contain 10 mM sodium phosphate buffer pH 7.0.

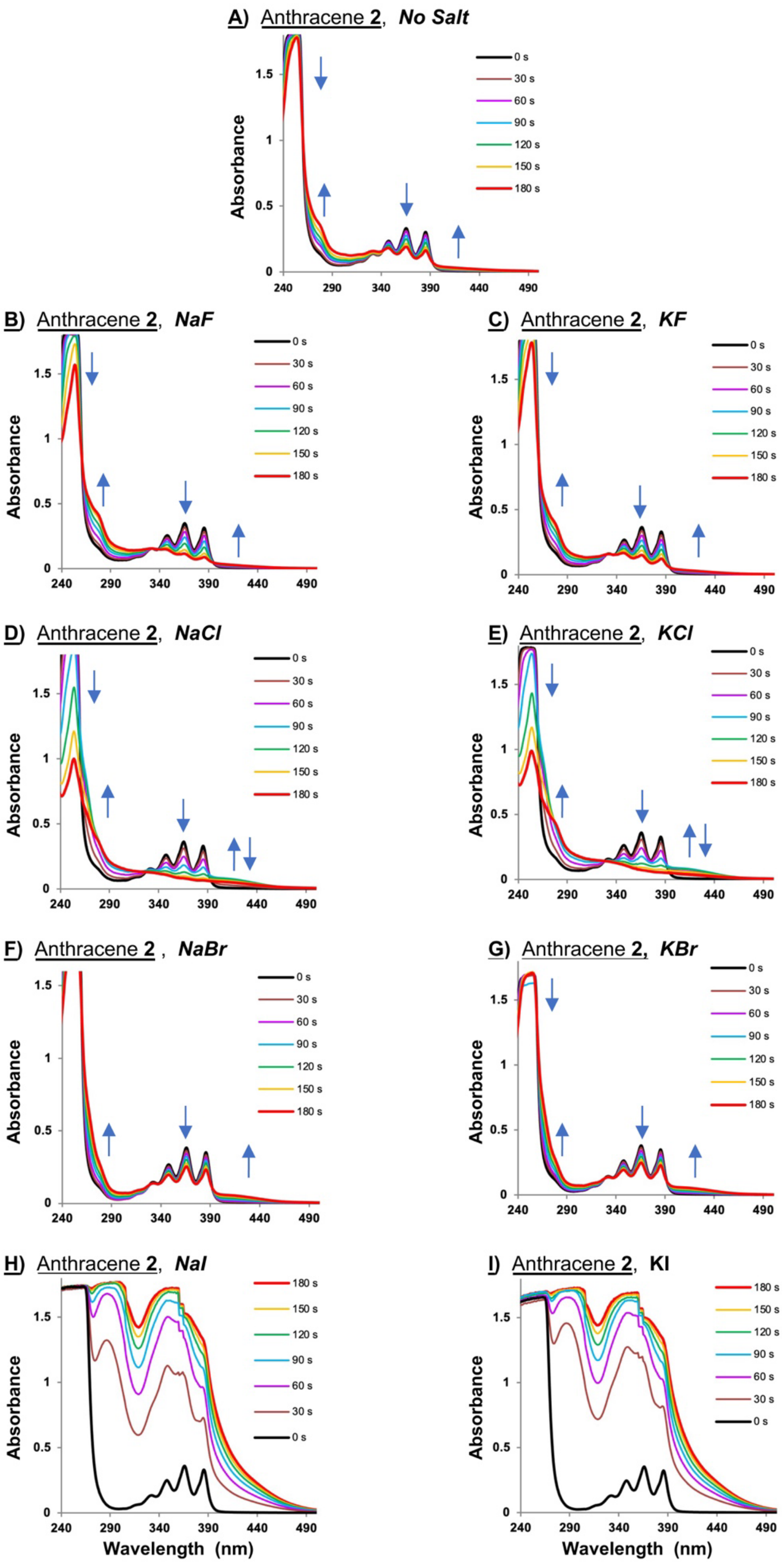

Figure S5. Representative spectra showing photo-induced degradation of 50 μM of N^1-(anthracen-9-ylmethyl)ethane-1,2-diaminium dichloride **2** in the presence and absence of 400 mM of halide salts in 10 mM sodium phosphate buffer pH 7.0. All samples were irradiated for time intervals from 0 s up to 180 s in a ventilated Rayonet photo-chemical reactor fitted with 8 RPR-3500 Å, 24 W lamps.

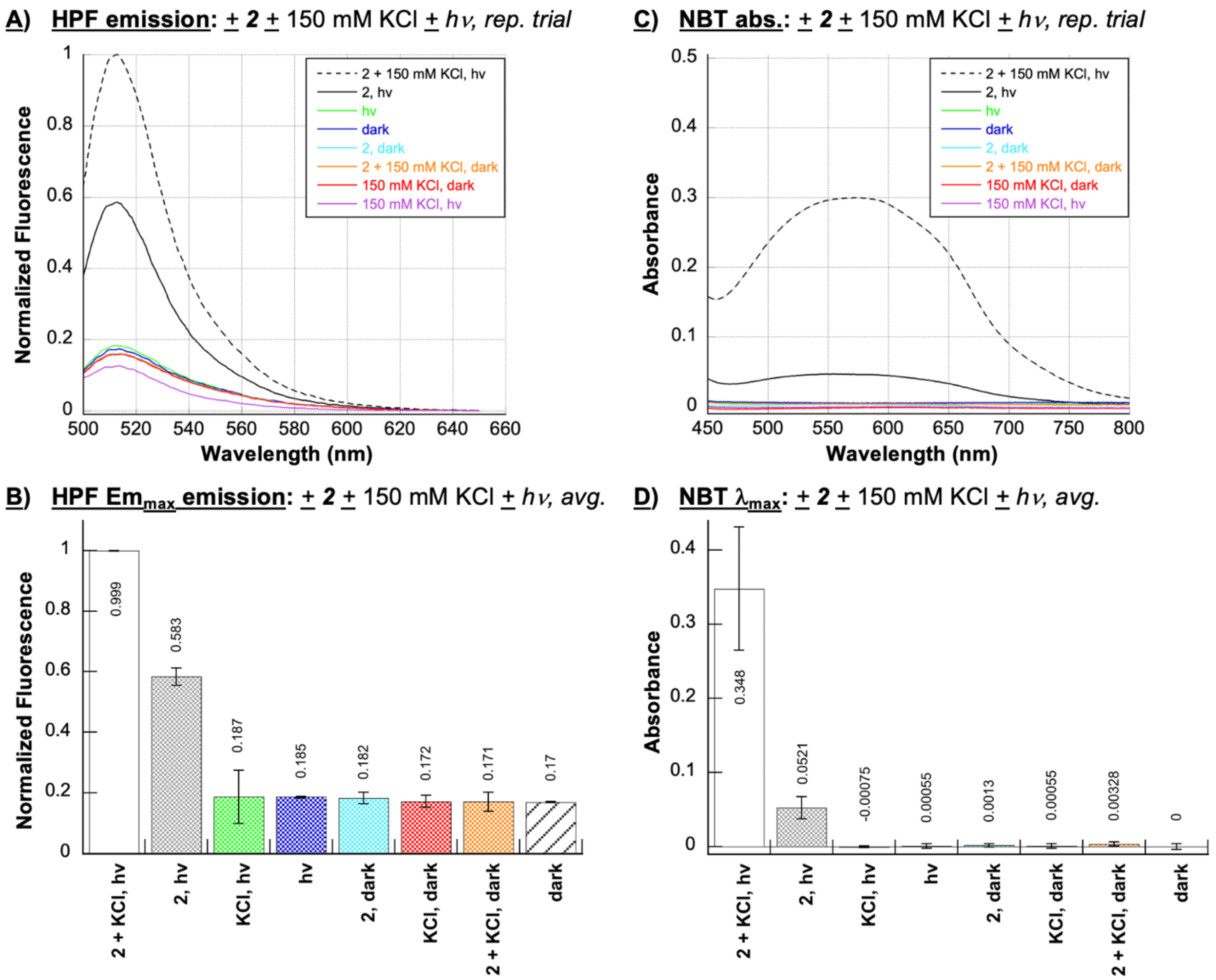

Figure S6. **HPF** **A)** Representative, normalized hydroxyphenyl fluorescein (HPF) emission spectra of 3 μM HPF and 10 mM sodium phosphate buffer pH 7.0 in the presence and absence of 1 μM of **2** and 150 mM of KCl (no DNA). Samples were irradiated for 7 min at 350 nm or kept in the dark. HPF emission spectra were then recorded from 500 to 650 nm at an excitation wavelength of 490 nm. **B**) Normalized fluorescence emission at 512.5 nm averaged over two or four trials for samples without and with **2**, respectively. Error bars represent standard deviation. **NBT** **C**) Representative nitro blue tetrazolium (NBT) absorption spectra of 16 μM of NBT and 10 mM sodium phosphate buffer pH 7.0 in the presence and absence of 50 μM of **2** and 150 mM of KCl (no DNA). Samples were irradiated for 5 min at 350 nm or kept in the dark. NBT absorption spectra were then recorded from 450 to 800 nm. **D**) Absorption at NBT λ_{max} (570 nm) averaged over two to four or four trials for samples without and with **2**, respectively. Error bars represent standard deviation.

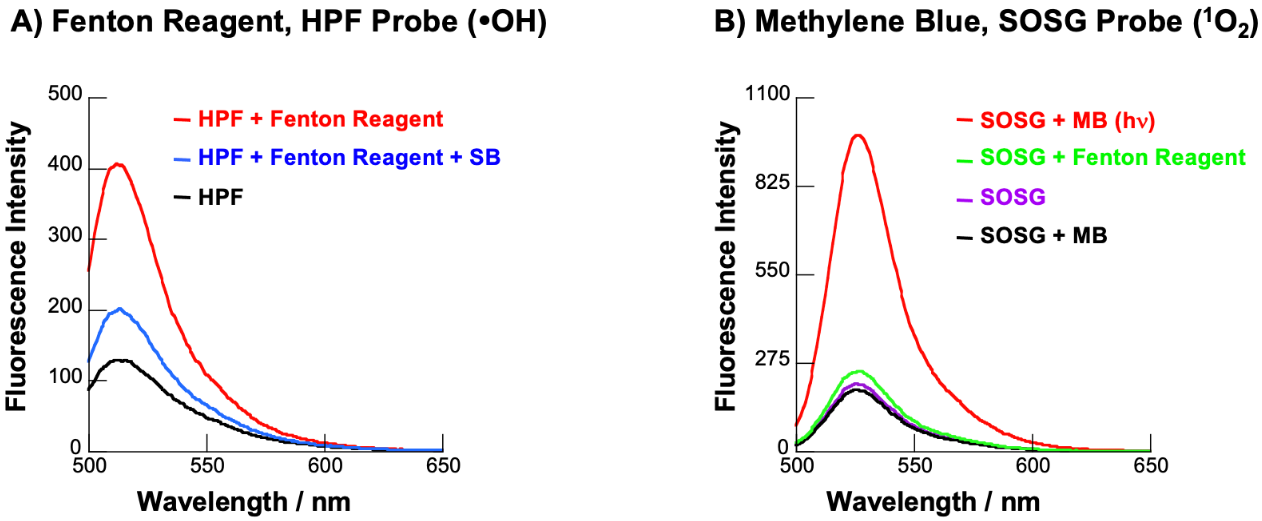

Figure S7. Fluorescence emission spectra recorded at 22 ºC of: **A**) 3 µM HPF in the absence and presence of either 10 µM ammonium iron(II) sulphate + 10 µM H_2O_2 (= 10 µM Fenton reagent) or 10 µM of Fenton reagent and 100 mM sodium benzoate (SB); **B**) 0.75 µM Singlet Oxygen Sensor Green® (SOSG) in the absence and presence of either 10 µM Fenton reagent, 1 µM methylene blue, or 1 µM methylene blue irradiated for 2 s with a 638 nm LED laser (hν, 2.8 W/cm^2, Laserland). All samples contained 10 mM sodium phosphate buffer pH 7.0. This figure was reproduced from Basnet, *et al.,* 2019[1] with permission from the Royal Society of Chemistry.

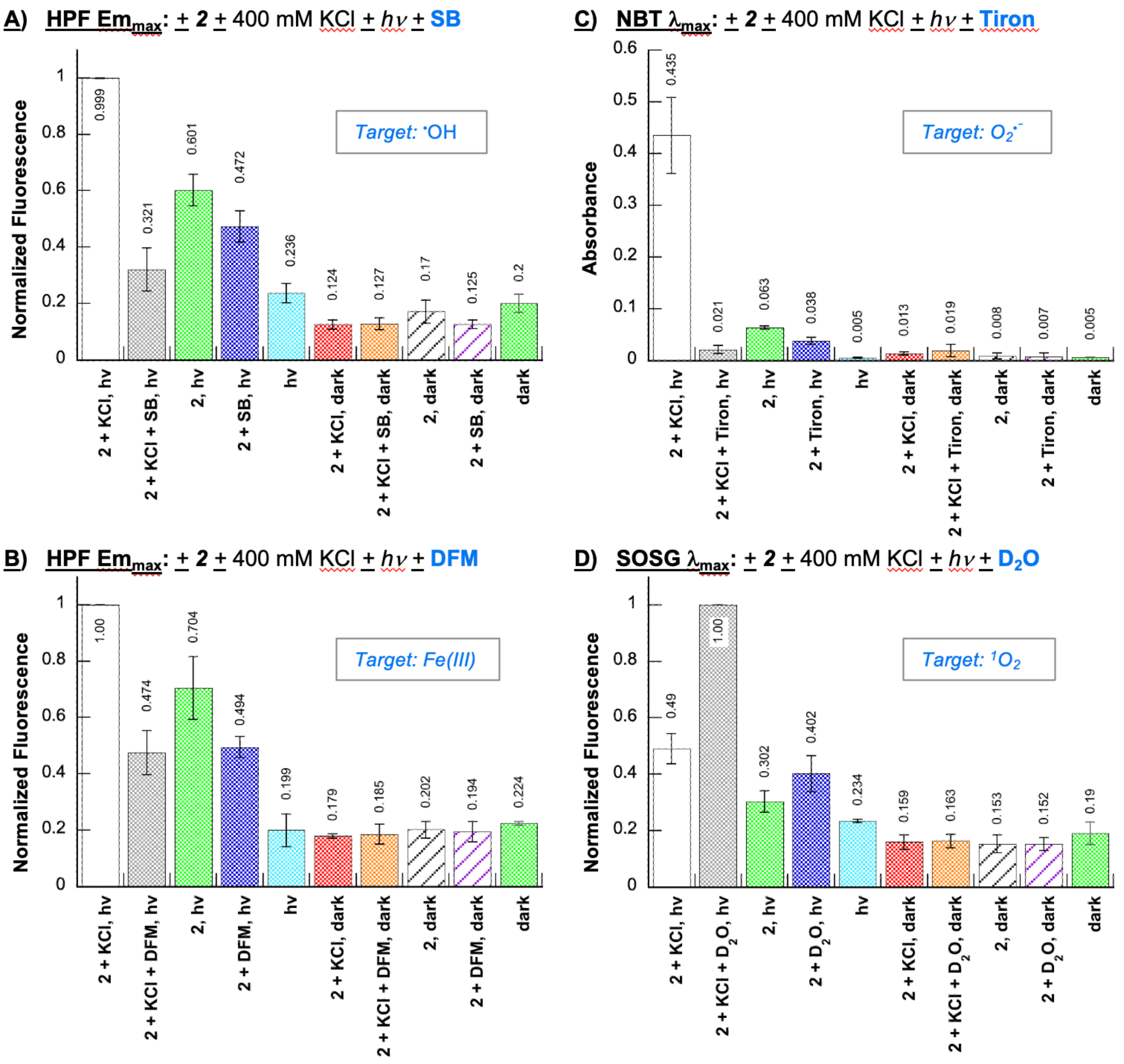

Figure S8. Reactions containing 10 mM sodium phosphate buffer pH 7.0 and a probe, either 3 µM of HPF, 16 µM of NBT, or 1 µM of SOSG were irradiated for 0, 5 (NBT), or 7 min (HPF, SOSG) at 350 nm in the presence and absence of 1 µM (HPF, SOSG) or 50 µM (NBT) of **2,** 400 mM of KCl, and a chemical additive: **A)** 100 mM of sodium benzoate (SB) to detect hydroxyl radicals; **B)** 5 mM of deferoxamine mesylate (DFM) to chelate Fe(III)**; C)** 1 mM of disodium 4,5-dihydroxy-1,3-benzenedisulfonate (Tiron) to scavenge superoxide anion radicals; and **D)** 90% *(v/v)* D_2O to extend the lifetime of singlet oxygen. Emission spectra were then recorded from 500 to 650 nm at excitation wavelengths of 490 nm (HPF) or 500 nm (SOSG). NBT absorption spectra were acquired from 450 to 800 nm. The following data were then averaged over 2 to 5 trials: **A)** and **B)** normalized fluorescence emission at 512.5 nm; **C)** absorption at 570 nm; **D)** normalized fluorescence emission at 526.5 nm. Error bars represent standard deviation.

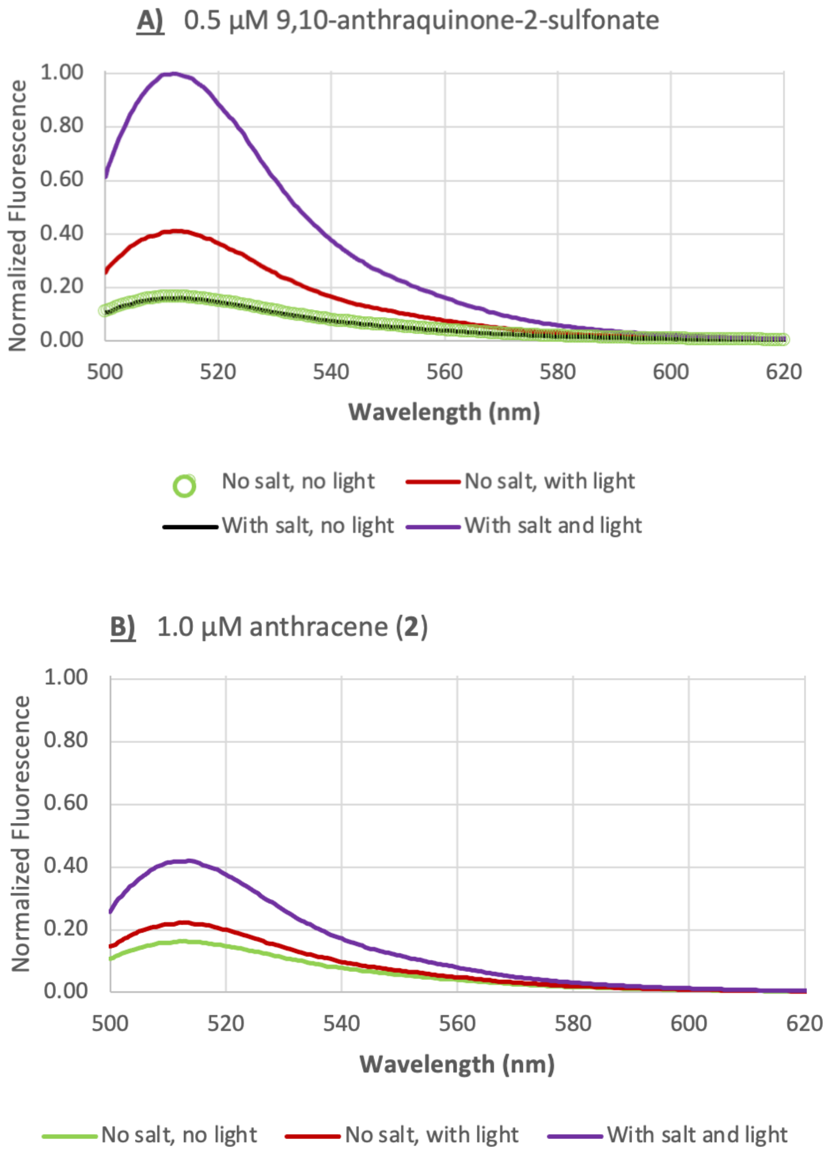

Figure S9. Normalized fluorescence emission spectra of hydroxyphenyl fluorescein (HPF). All samples contained either **A**) 0.5 µM of 9,10-anthraquinone-2-sulfonate or **B**) 1.0 µM of N^1-(anthracen-9-ylmethyl)ethane-1,2-diaminium dichloride **2**, 3 µM HPF, and 10 mM sodium phosphate buffer pH 7.0 in the absence and presence of 400 mM of KCl. The samples were irradiated for 60 s in a ventilated Rayonet photo-chemical reactor fitted with 8 RPR-3500 Å, 24 W lamps. The HPF fluorescence emission spectra were recorded from 500 to 650 nm using an excitation wavelength of 490 nm.

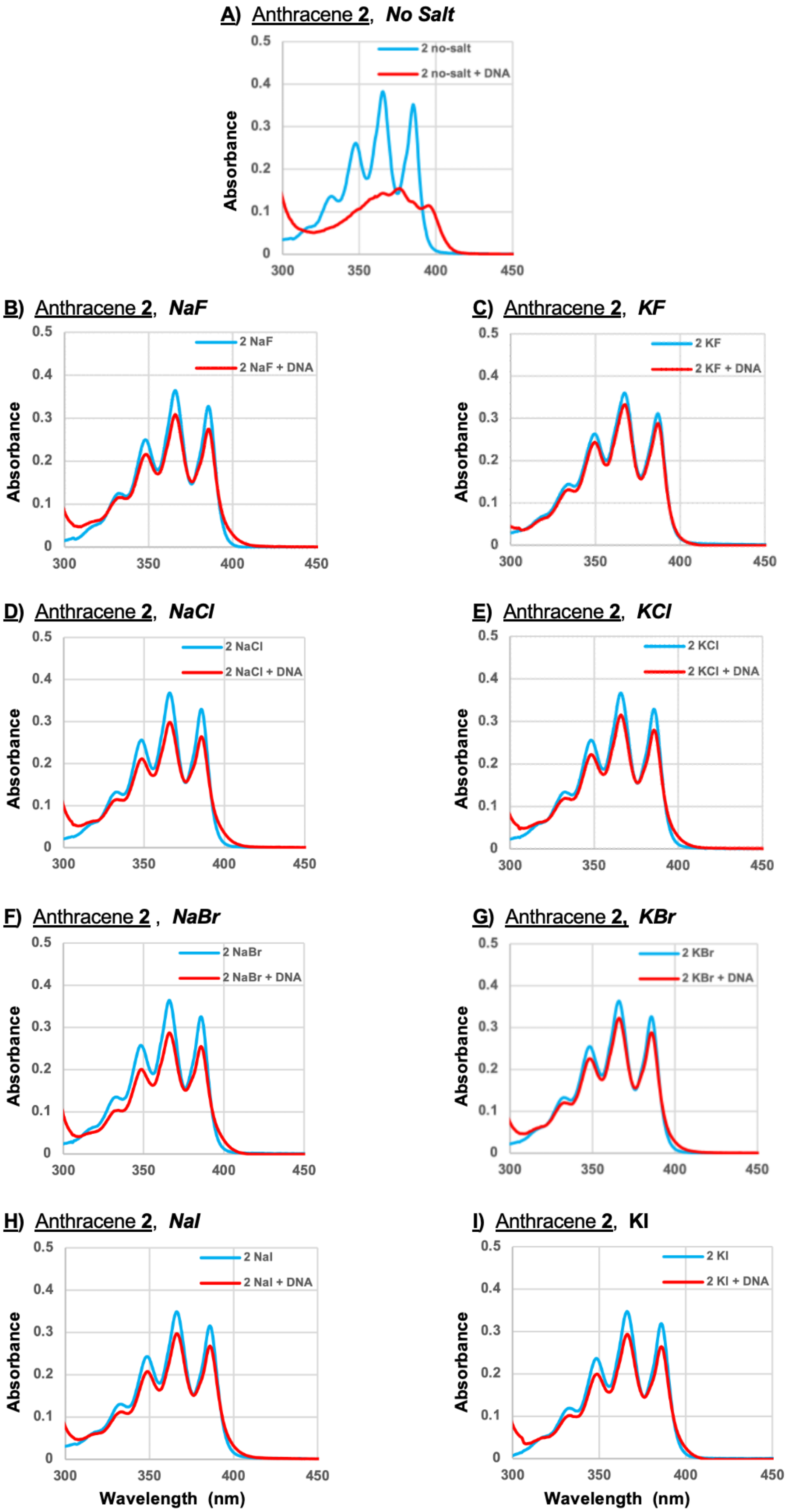

Figure S10. UV-visible absorbance spectra of 50 μM of N^1-(anthracen-9-ylmethyl)ethane-1,2-diaminium dichloride **2** in the presence and absence of 200 μM bp of CT DNA with either 0 mM **A**) or 400 mM of a halide salt: **B**) NaF, **C**) KF, **D**) NaCl, **E**) KCl, **F**) NaBr, **G**) KBr, **H**) NaI and **I**) KI.

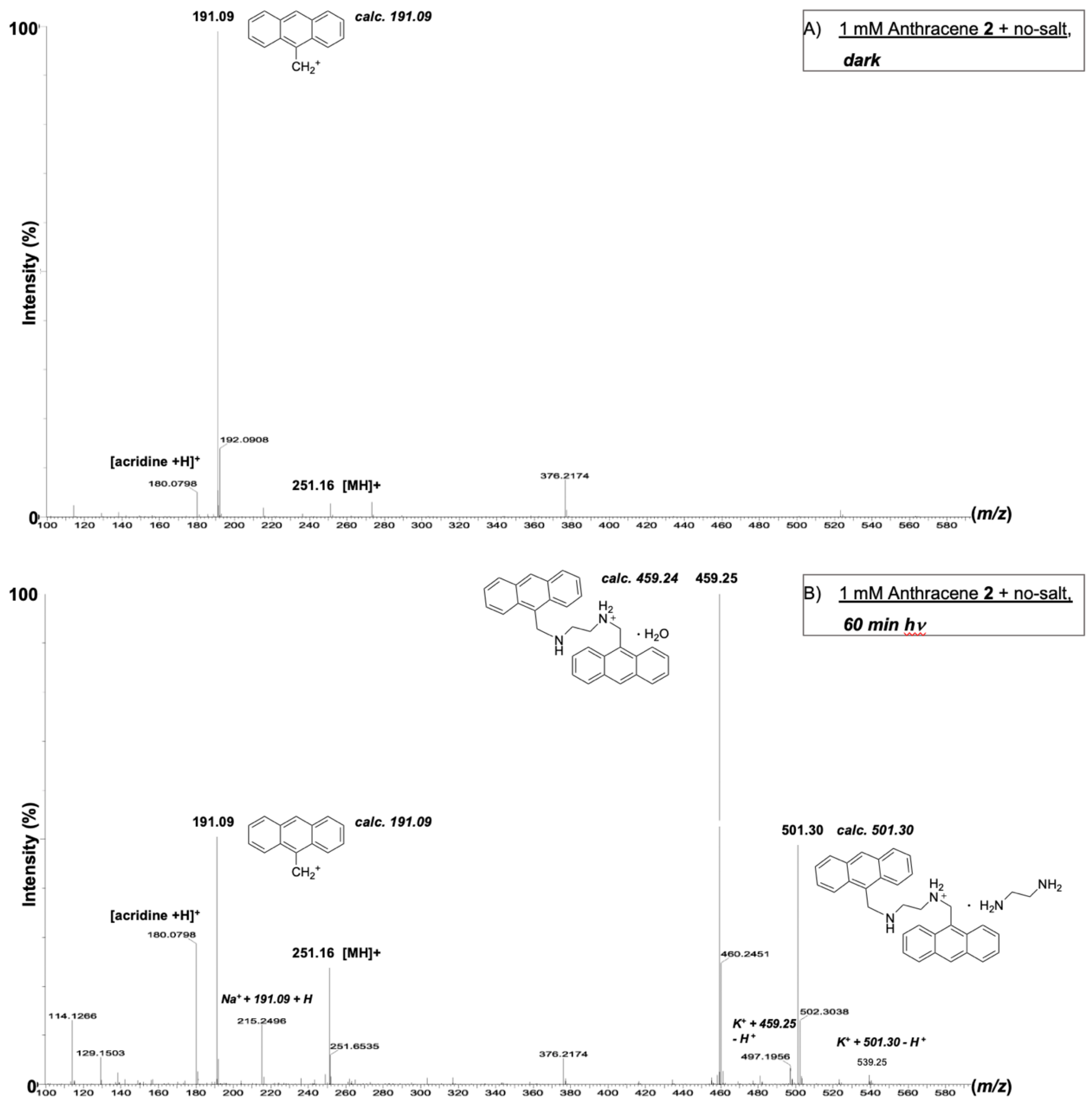

Figure S11. ESI mass spectra of N^1-(anthracen-9-ylmethyl)ethane-1,2-diaminium dichloride **2**. Samples **A**) and **B**) contained 1 mM of **2** and were respectively irradiated with 350 nm UV light for 0 min and 60 min (no-salt, 10 mM ammonium formate buffer pH 7.0). A 1 mM acridine standard was added at a one-to-one ratio and the resulting solution was diluted 100-fold in 10 mM ammonium formate buffer pH 7.0. ESI analyses were then performed on a Waters Xevo G2-XS Mass Spectrometer equipped with an electrospray ionization source in a positive ion mode.

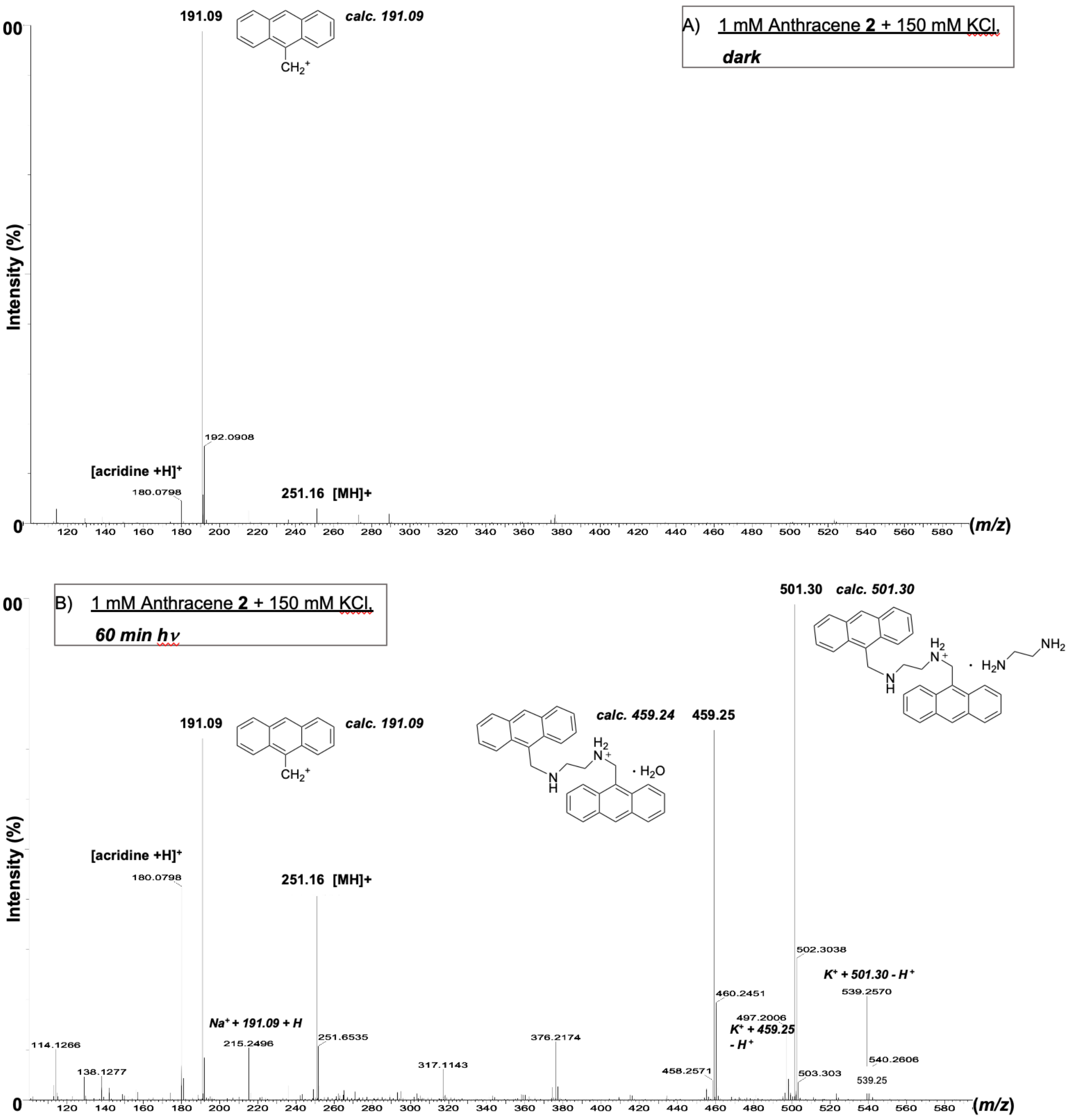

Figure S12. ESI-MS spectra of N^1-(anthracen-9-ylmethyl)ethane-1,2-diaminium dichloride **2**. Samples **A**) and **B**) contained 1 mM of **2** and were respectively irradiated with the 350 nm UV light source for 0 min and 60 min (150 mM KCl, 10 mM ammonium formate buffer pH 7.0). A 1 mM acridine standard was added at a one-to-one ratio and the resulting solution was diluted 100-fold in 10 mM ammonium formate buffer pH 7.0. ESI analyses were then performed on a Waters Xevo G2-XS Mass Spectrometer equipped with an electrospray ionization source in a positive ion mode.

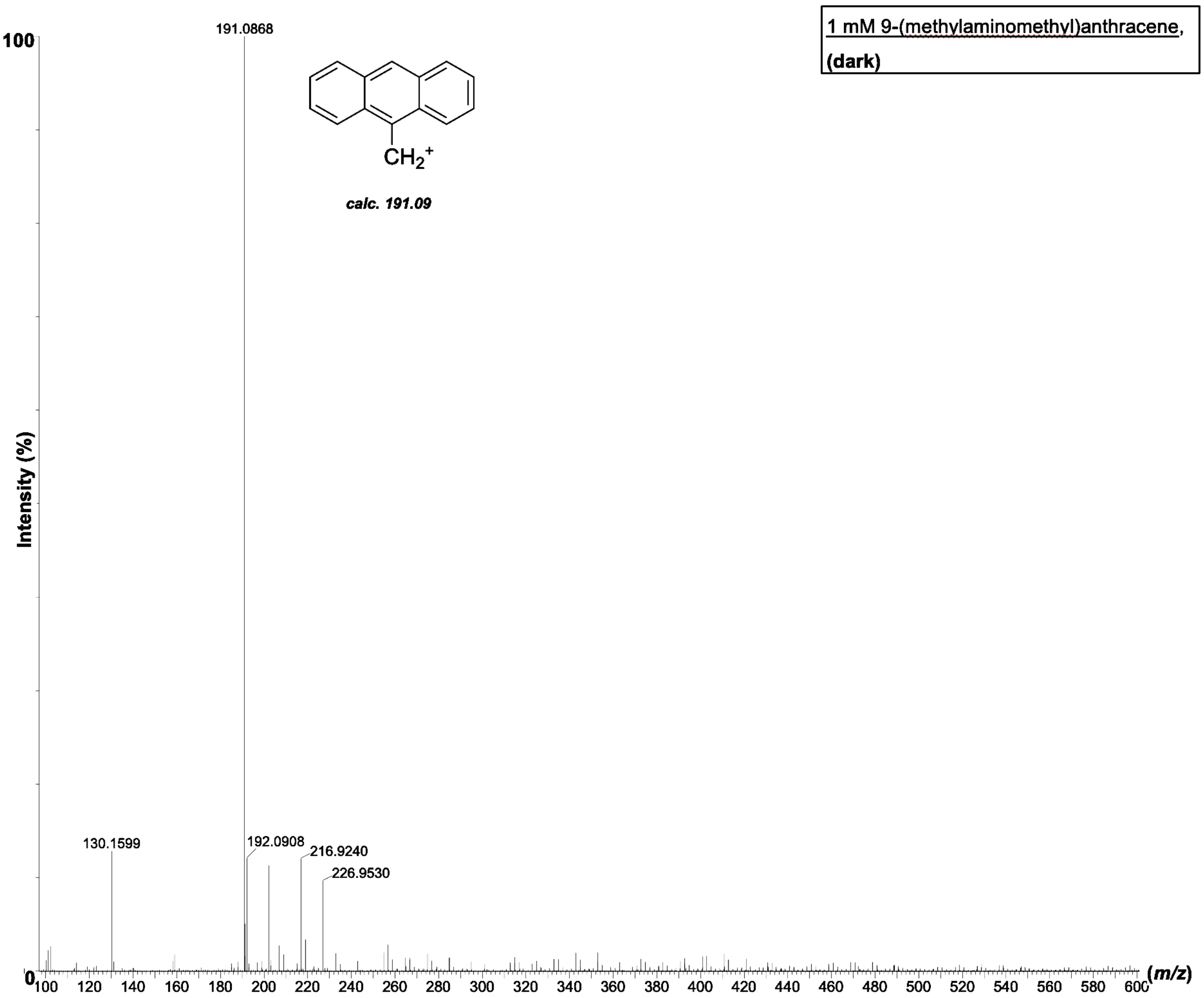

Figure S13. ESI-MS spectrum of commercially available 9-(methylaminomethyl)anthracene (Figure S1). The sample, which contained 1 mM of the anthracene and 10 mM ammonium formate buffer pH 7.0, was diluted 100-fold in 10 mM ammonium formate buffer pH 7.0. ESI analysis was then performed on a Waters Xevo G2-XS Mass Spectrometer equipped with an electrospray ionization source in a positive ion mode. The anthracen-9-yl methylium ESI-MS fragmentation product (*m/z* = 191.01) of the anthracene is the major peak in the spectrum. The parent ion of 9-(methylaminomethyl)anthracene ($[MH]^+$), with an empirical formula of $C_{16}H_{16}N$ and a calculated exact mass *m/z* ratio of 222.128, is not visible.

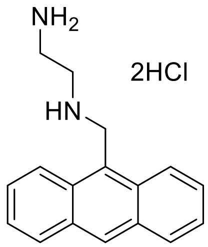

2

Figure S14. Analytical data of N^1-(anthracen-9-ylmethyl)ethane-1,2-diaminium dichloride **2**. ^{1}H-NMR (400 MHz, D_2O) δ 8.37 (s, 1H), 8.03 (d, J = 8.8 Hz, 2H), 7.93 (d, J = 8.4 Hz, 2H), 7.59 – 7.53 (m, 2H), 7.49 – 7.43 (m, 2H), 4.96 (s, 2H), 3.44 (t, J = 7.2 Hz, 2H), 3.23 (dd, J = 9.4, 4.9 Hz, 2H). ^{13}C-NMR (101 MHz, D_2O) δ 130.37, 129.85, 129.28, 127.60, 125.31, 122.23, 119.35, 44.15, 43.18, 35.35. MS (ESI) *m/z*: calcd for: $C_{17}H_{19}N_2$, 251.35, found 251.15.

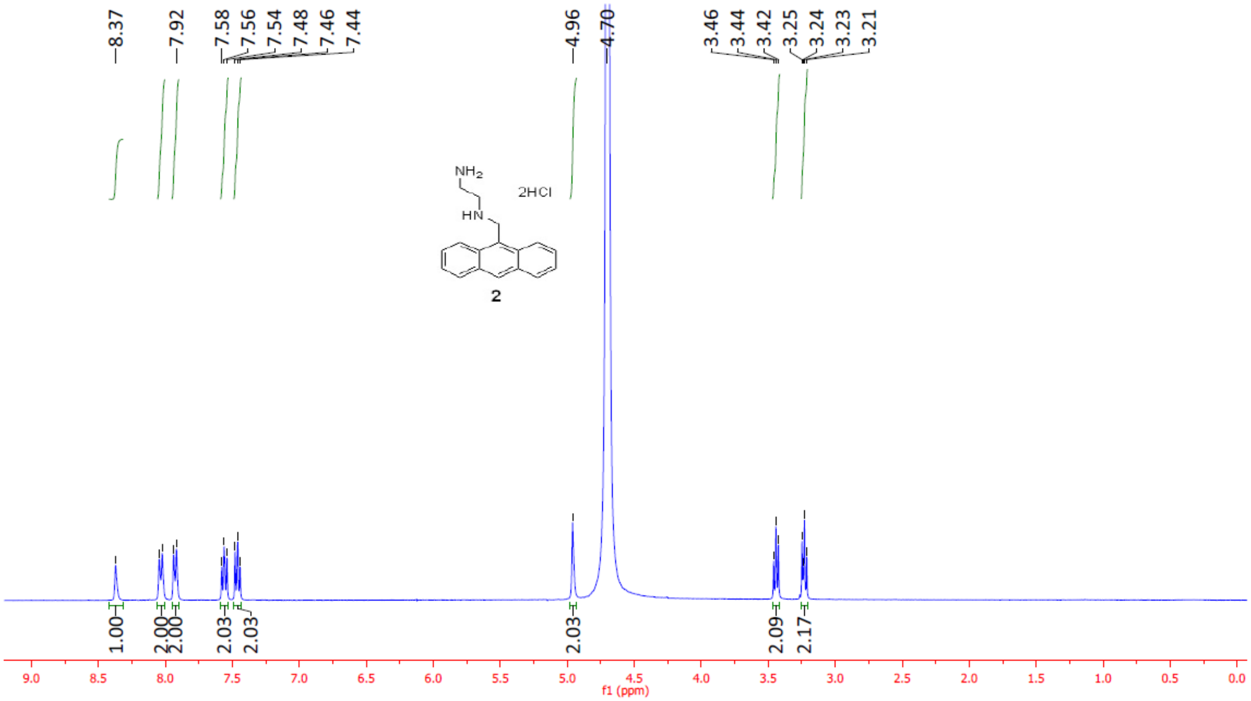

Figure S15. ^{1}H-NMR of N^1-(anthracen-9-ylmethyl)ethane-1,2-diaminium dichloride.

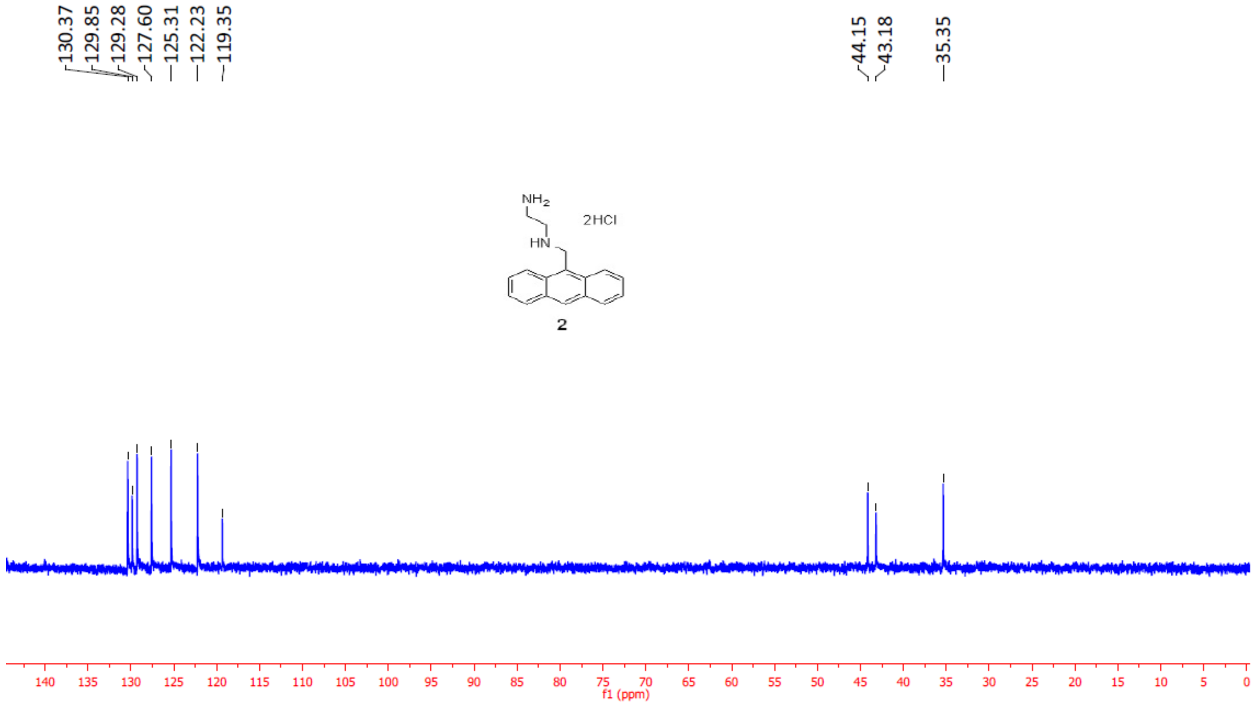

Figure S16. ^{13}C-NMR of N^1-(anthracen-9-ylmethyl)ethane-1,2-diaminium dichloride.

Reference:

(1) Basnet, K.; Fatemipouya, T.; St Lorenz, A.; Nguyen, M.; Taratula, O.; Henary, M.; Grant, K. B. Single photon DNA photocleavage at 830 nm by quinoline dicarbocyanine dyes. *Chem. Commun.* **2019**, *55*, 12667-12670.
